# The *bantam* microRNA acts through Numb to exert cell growth control and feedback regulation of Notch in tumor-forming stem cells in the *Drosophila* brain

**DOI:** 10.1371/journal.pgen.1006785

**Published:** 2017-05-17

**Authors:** Yen-Chi Wu, Kyu-Sun Lee, Yan Song, Stephan Gehrke, Bingwei Lu

**Affiliations:** 1 Department of Pathology, Stanford University School of Medicine, Stanford, CA, United States of America; 2 BioNanotechnology Research Center, Korea Research Institute of Bioscience and Biotechnology, Daejeon, Korea; 3 School of Life Sciences and Peking-Tsinghua Center for Life Sciences, Peking University, Beijing, China; RIKEN Brain Science Institute, JAPAN

## Abstract

Notch (N) signaling is central to the self-renewal of neural stem cells (NSCs) and other tissue stem cells. Its deregulation compromises tissue homeostasis and contributes to tumorigenesis and other diseases. How N regulates stem cell behavior in health and disease is not well understood. Here we show that N regulates *bantam* (*ban*) microRNA to impact cell growth, a process key to NSC maintenance and particularly relied upon by tumor-forming cancer stem cells. Notch signaling directly regulates *ban* expression at the transcriptional level, and *ban* in turn feedback regulates N activity through negative regulation of the Notch inhibitor Numb. This feedback regulatory mechanism helps maintain the robustness of N signaling activity and NSC fate. Moreover, we show that a Numb-Myc axis mediates the effects of *ban* on nucleolar and cellular growth independently or downstream of N. Our results highlight intricate transcriptional as well as translational control mechanisms and feedback regulation in the N signaling network, with important implications for NSC biology and cancer biology.

## Author summary

Stem cells are functional units in the development, maintenance, and regeneration of tissues in multicellular organisms. Defects in stem cell regulation can compromise tissue homeostasis and result in proliferative or degenerative diseases. Our understanding of the molecular and cellular mechanisms regulating the *in vivo* behavior of stem cells is still incomplete. The *Drosophila* central nervous system neural stem cells called neuroblasts have offered an excellent model system for uncovering key mechanisms and player involved in stem cell regulation. Previous genetic studies have uncovered the evolutionarily conserved Numb-N signaling pathway that regulates the self-renewal vs. differentiation choices of the cell fates of NSCs during their asymmetric division. Our understanding of how Numb-N signaling regulates NSC fate is still rudimentary. Recent studies have implicated the involvement of microRNAs in stem cell regulation in both mammalian and *Drosophila* systems. But the molecular mechanism and logic of miRNA action remain to be delineated. In this study we show that the *bantam* microRNA is a direct transcriptional target of the N signaling pathway, and that *bantam* feedback regulates N by negatively regulating the expression of Numb, an inhibitor of N. This feedback regulation of N helps maintain the robustness of NSC fate. We further show that *bantam* also impinges on a Numb-Myc axis of cell growth regulation, apparently in a N-independent manner. Together, our results highlight the importance of both transcriptional and translational control mechanisms in NSC regulation by the N signaling network. These findings have important implication for our understanding of the basic biology of NSCs and the therapeutic intervention of N-induced cancers.

## Introduction

Balancing self-renewal with differentiation is a key property of all stem cells [1–3]. Tipping such balance can have detrimental consequences, resulting in lineage depletion or tumorigenesis. N signaling is critically required for lineage homeostasis of both *Drosophila* and mammalian NSCs [3–5]. In the *Drosophila* larval central brain, there are two different types of neuroblast (NB) lineages, the type I and type II NBs. N signaling appears to be dispensable for the homeostasis of type I NB lineages. In contrast, in the type II NB lineages, which differs from type I NB lineages by possessing transit-amplifying intermediate progenitors (IPs) and are hierarchically similar to mammalian NSCs, impaired N signaling leads to NB loss whereas N hyperactivation causes the dedifferentiation of IPs into cancer stem cell (CSC)-like tumor-initiating NBs [6–8]. Dedifferentiation has also been recognized as a key mechanism in tumorigenesis in mammals, highlighting the relevance of *Drosophila* type II NBs to the understanding of human cancer biology.

The mechanism by which N signaling maintains NB lineage homeostasis is not well defined. Cell growth regulation has recently been implicated as a key mechanism by which N maintains NSCs [7], and is particularly relied upon by CSC-like NSCs [7, 9]. Understanding how N signaling regulates the growth and maintenance of normal NSCs and CSC-like NSCs will therefore have important implications for NSC biology and cancer biology. MicroRNAs are non-coding mRNAs that regulate gene expression by base-pairing with target mRNAs to inhibit their translation or stability. The mode of microRNA action in regulating gene expression tends to be fine-tuning instead of on-or-off, making them excellent candidate players in the maintenance of the robustness of cell fates and tissue homeostasis. The microRNA pathway has emerged as a fundamental gene regulatory pathway with important roles in cell metabolism, proliferation, differentiation, and survival [10–15]. Although recent studies have implicated the involvement of microRNAs in stem cell regulation in various organisms, the molecular mechanisms and logic of microRNA action remain to be elucidated.

In this study we set out to examine the role of the *bantam* (*ban*) microRNA in the regulation of NB homeostasis in the *Drosophila* brain. We show that *ban* is a direct transcriptional target of the N signaling pathway, and that *ban* feedback regulates N through negative regulation of its target mRNA *numb*, which encodes an inhibitor of N. We show that this feedback regulation of N helps maintain the robustness of NB cell fate. Our results further show that *ban* also impinges on a Numb-Myc axis of cell growth regulation, apparently in a N-independent manner, thus revealing novel mechanisms in NSC regulation by the N signaling network. These findings have important implications for both the basic biology of NSCs and the therapeutic intervention of cancers caused by deregulated Numb-N signaling.

## Results

**Fig 1:**
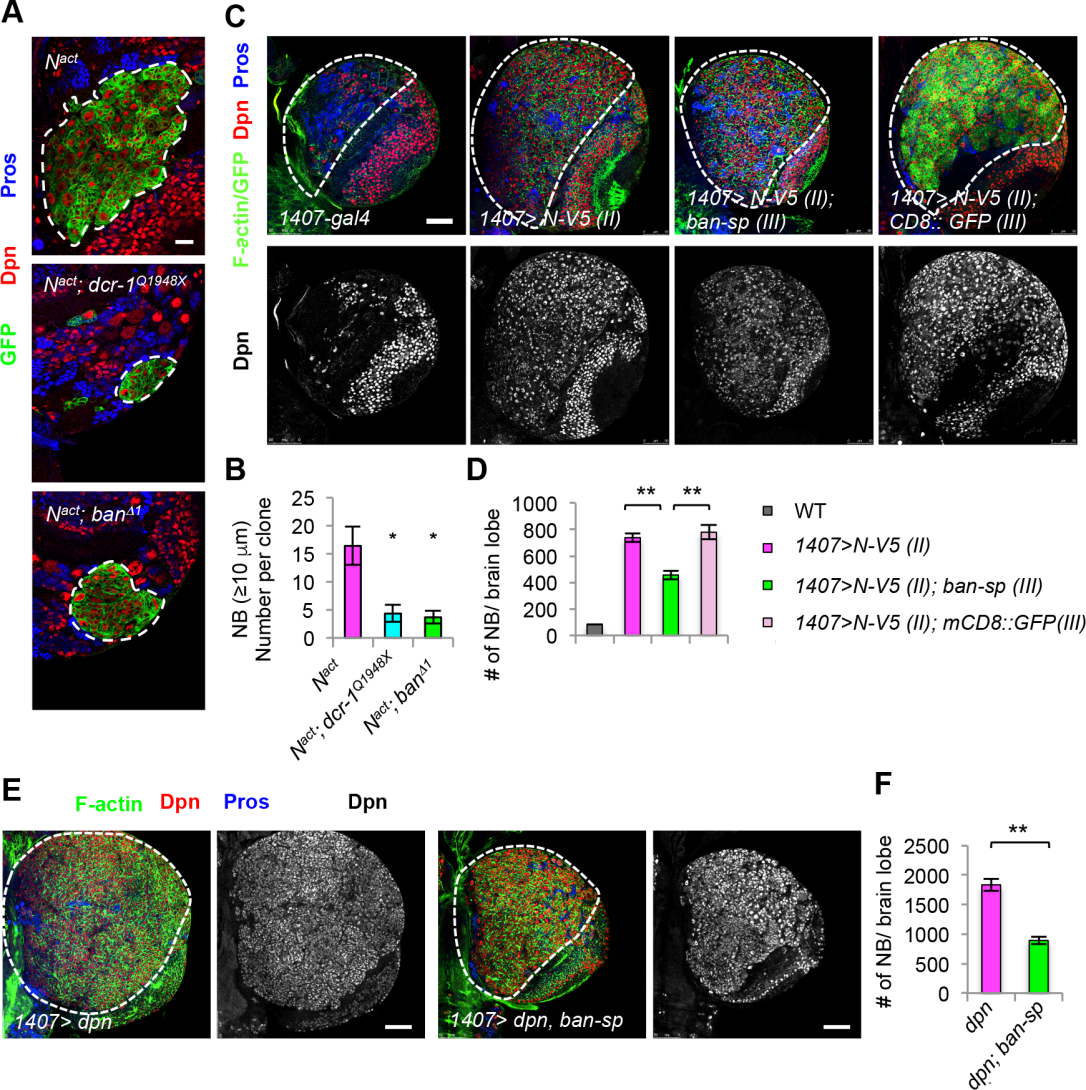
The growth regulator *ban* is preferentially required for CSC-like NB proliferation and maintenance. (**A**) Clonal analysis of NBs overexpressing *N*^*act*^ in control, *dicer-1*^*Q1948X*^, *or ban*^*Δ1*^ backgrounds. Larval brains of different genotypes at 96 h ALH were immunostained for Dpn and Pros. Type II NB MARCM clones are marked with GFP and outlined with white dashed lines. (**B**) Quantification of NB number in samples from **A**. *, *p***<**0.001 in Student’s *t*-test; n **=** 4–10 clones. (**C**) Effect of *ban* inhibition by *ban-sp* on N-induced NB overproliferation. Larval brains were stained for F-actin (Green, cell cortex), Dpn (red, NBs and mature IPs), and Pros (GMCs and neurons). The central brain area is outlined with a bold white dashed line, and the Dpn^+^ NBs within this area were quantified. (**D**) Quantification of data from **C**. **, *p*<0.0001; n = 8–10 brains. (**E**) Effects of *ban* inhibition by *ban-sp* on Dpn OE-induced type II lineage NB overproliferation. (**F**) Quantification of number of NBs in **E**. **, *p***<**0.00001; n **=** 8 brains. Scale bars: **A**, 20 μm; **C**, **E**, 50 μm.

**Fig 2:**
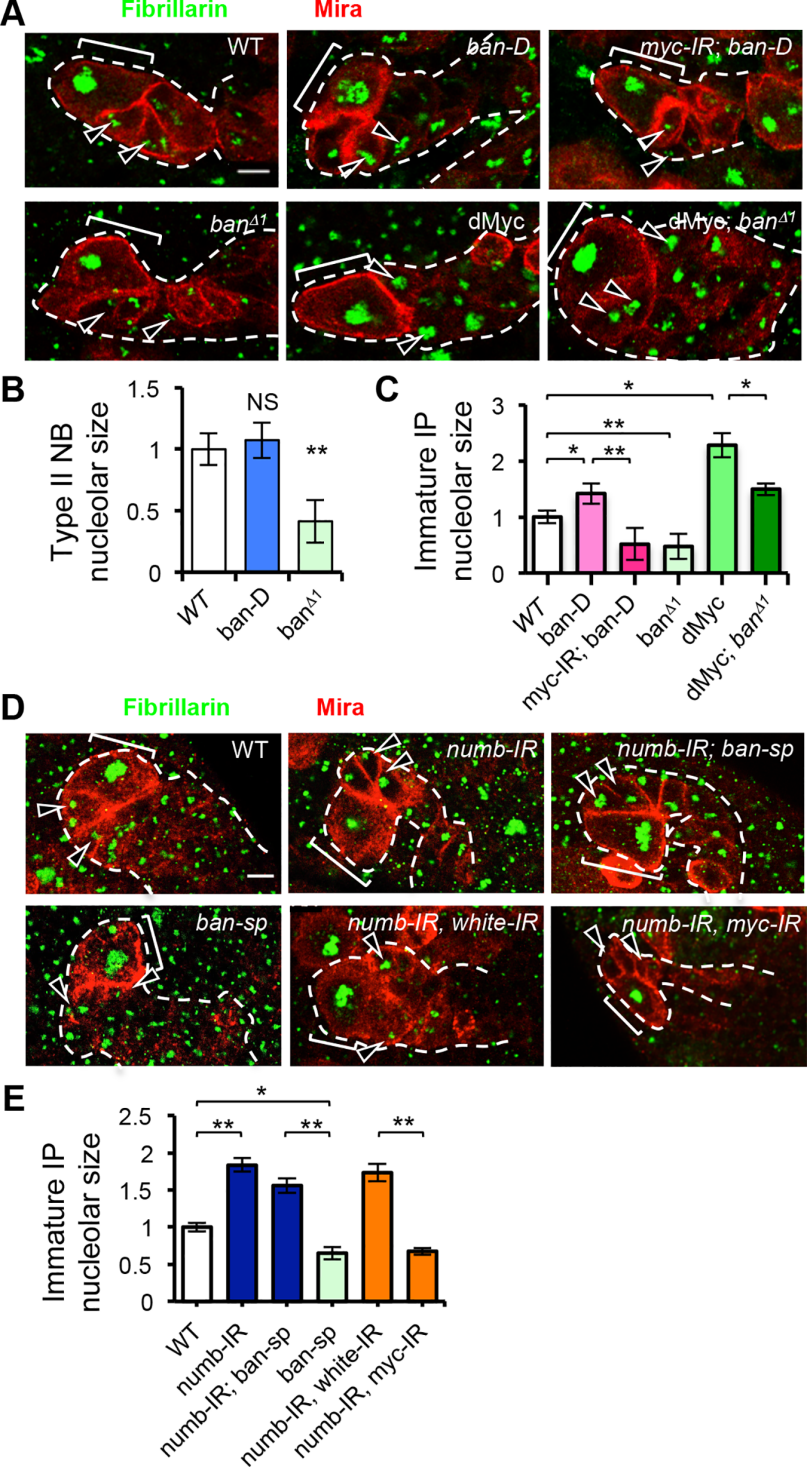
Regulation of nucleolar growth by *ban* and Numb in type II NB lineages. (**A**) Genetic interaction between *ban* and *myc* in nucleolar size regulation. Green, Fibrillarin; Red, Miranda; Bracket, NB. Arrowheads: nucleoli of immature IPs. (**B**) Quantification of effect on nucleolar size by *ban* GOF (*ban*-D OE) and *ban* LOF (*ban*^*Δ1*^) in type II NBs from **A**. *, *p*< 0.005; NS, not significant; n = 10–12 type II NB lineages/genotype. (**C**) Quantification of nucleolar size of immature IPs in type II NB lineages from **A**. *, *p*< 0.001; **, *P*< 0.00001; n = 9–12 type II NB lineages/genotype. (***D***) Genetic interactions between Numb and *ban* and between Numb and Myc in nucleolar growth regulation. (**E**) Quantification of nucleolar size of immature IPs in type II NB lineages from **D**. *, *p***<**0.001, **, *p***<**0.00001; n **=** 8 type II NB lineages /genotype. Scale bars: **A**, **D**, 5 μm.

To identify new players in the N signaling network important for NSC and CSC-like growth, we tested the microRNA pathway. In the fly larval central brain, N signaling is normally required for the maintenance of type II but not type I NBs, and N hyperactivation results in the formation of CSC-like NB within the type II but not type I NB lineages [6–8]. We used clonal overexpression (OE) of N intracellular domain (N-intra), an activated form of N, to induce ectopic formation and overproliferation of type II NBs and ensuing tumorous brain growth (Fig 1A and 1B). Inactivation of Dicer-1 (Dcr-1), a key component of the miRNA pathway [16], effectively rescued N-intra induced ectopic NB formation and tumorous growth, supporting a critical role for miRNA in N-regulated NSC lineage homeostasis (Fig 1A and 1B). Given the role of *ban* miRNA in controlling tissue growth, cell proliferation, and survival [17, 18], we tested its involvement in NSC regulation by N. Loss of *ban* function as in *ban*^*Δ1*^ null mutant had similar effect as *dcr-1* mutation in rescuing N-intra induced ectopic NB formation (Fig 1A and 1B), implicating *ban* as a key miRNA influencing NB homeostasis. To further confirm these results, we used a transgene overexpressing *ban*-sponge (*ban-sp*), which could effectively interfere with *ban* function [19]. CSC-like ectopic NB proliferation induced by OE of N [7, 9] was partially blocked by *ban-sp* (Fig 1C and 1D). Conversely, *ban* OE enhanced the N OE effect (S1 Fig). Overexpression of Dpn, a putative effector of the N pathway in NBs, caused the formation of CSC-like NB within the type II but not type I NB lineages [20, 21]. NB overproliferation caused by Dpn-OE was also attenuated by *ban-sp* (Fig 1E and 1F). Intriguingly, in *ban*^*Δ1*^ mutant type II NB clones without N OE, the parental NBs were preserved (Figs 2A and S2E), suggesting that *ban* is not essential for NB formation or maintenance under normal condition. The number of IPs, however, is reduced in *ban*^*Δ1*^ mutant type II NB clones (S2F Fig). These results are consistent with a recent report [18], which showed that both type I and type II NBs are reduced in *ban* mutant brains. However, in clonal analysis, each *ban* mutant clone still contains a NB with appropriate marker expression, albeit with reduced cell size [18]. These results suggest that *ban* may act cell autonomously to regulate NB cell size, but its effect on NB number may be mediated by a non-autonomous mechanism. Together, these results suggest that *ban* is preferentially required for the formation and proliferation of CSC-like cells induced by N pathway hyperactivation.

We next tested whether cell growth regulation is a main mechanism by which *ban* mediates N effect on NSC/CSC regulation. Compared to WT NBs, *ban*^*Δ1*^ mutant NBs in NB clones are smaller in size, consistent with a previous report [18]. This is true in type I and type II NB lineages (S2A–S2C Fig). *ban*^*Δ1*^ mutant IPs in type II NB lineages are also smaller in size (S2D Fig). Conversely, overexpression of *ban* using a *UAS-ban-D* transgene increased the size of IPs, without obvious change of type II NB size (S2G–S2I Fig). These results support the notion that *ban* is involved in the growth control of NBs and IPs [18]. Previous studies demonstrated that nucleolar growth is a key aspect of cell growth in the dedifferentiation of IPs into ectopic NBs induced by N hyperactivation [7]. We found that the nucleolar sizes of both NBs and IPs in *ban*^*Δ1*^ type II NB lineages were smaller than WT (Fig 2A–2C). Conversely, when *ban* was overexpressed, the nucleolar size of IPs was increased (Fig 2A–2C). The growth regulator Myc is a key mediator of N-regulated nucleolar growth in NB lineages [7]. Knockdown of *dMyc* effectively attenuated *ban* OE induced nucleolar growth (Fig 2A and 2C), whereas dMyc OE rescued the nucleolar growth defect in *ban*^*Δ1*^ mutant (Fig 2A and 2C). As reported before [7], dMyc OE promotes nucleolar growth in IP but not NBs, whereas the depletion of dMyc leads to reduction of nucleolar size in both NBs and IPs. It appears that the nucleolar size in NBs has reached a limit making it hard for dMyc to further increase it. These results suggest that *ban* influences NB cell growth at least in part through Myc-mediated nucleolar growth, although we do not rule out the possibility that *ban* and dMyc may act in parallel to regulate nucleolar growth.

**Fig 3:**
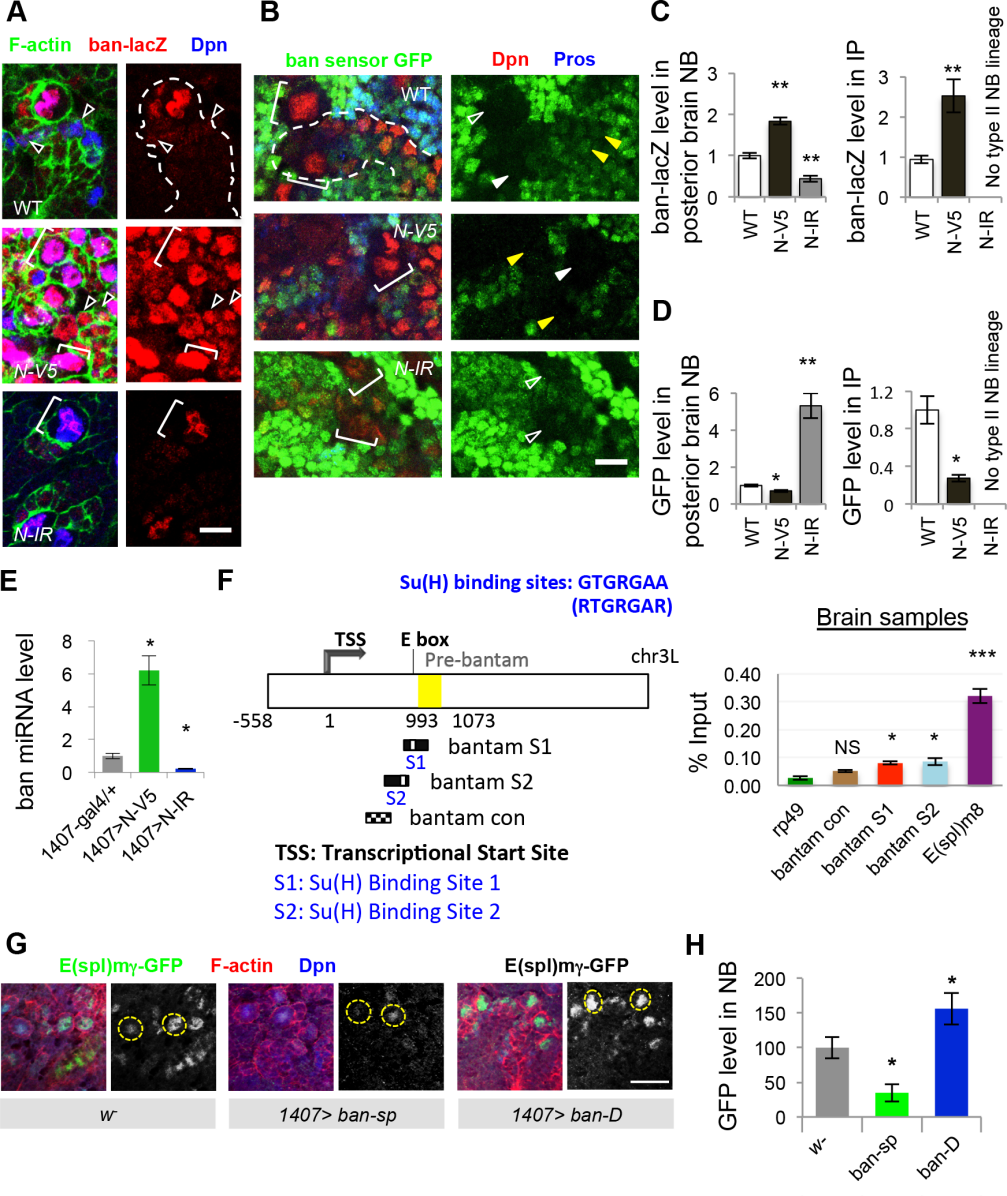
Regulation of *ban* expression and activity by N signaling and feedback regulation of N activity by *ban*. (**A**) Effects of N OE and N RNAi (N-IR) on *ban* expression as monitored with the *ban-lacZ* transcriptional reporter in type II NB lineages. N-V5 or N-IR transgene induction was carried out using a *1407*^*ts*^ system (*1407-GAL4*: *tub-GAL80*^*ts*^). Larva were shifted to 29°C at 24 hr ALH, and analyzed at late third instars. Green, F-actin; Red, LacZ; Blue, Dpn; Bracket, NB; Arrowheads, mature IPs. Note that N-IR brain has no type II NB lineages. (**B**) Effects of N OE and N-IR on *ban* activity as measured with *ban* GFP sensor expression. Green, GFP; Red, Dpn; Blue, Pros; Bracket, NBs; Open arrowhead, type I NB; Closed arrowhead and dotted outline, type II NB and its lineage; Yellow arrowhead, mature IP. (**C**) Quantification of *ban*-LacZ expression shown in **A**. n = 10–15 type I or II NBs from posterior brain /genotype (left), and n = 16–22 mature IPs/genotype (right). **, *p*<0.0005. (**D**) Quantification of *ban* GFP sensor expression. n = 5 type I or II NBs from posterior brain/genotype (left), and n = 5 mature IPs /genotype (right). *, *p*<0.005; **, *p*<0.0005. (**E**) Quantitative RT-PCR analysis of *ban* levels in third instar larval brains. *ban* levels were normalized to 2S rRNA. *, *p*<0.005; mean ± SEM, n = 3 repeats. (**F**) ChIP analysis of Su(H) binding to genomic DNA in *ban* locus. (Left) Schematic drawing of the *ban* locus. The position of pre-miRNA sequence in yellow box is between 993–1073 nucleotides (counting from transcription start site at position 1). Sequences containing two putative Su(H)-binding sites (S1 and S2) that matched the consensus sequence RTGRGAA, and a control sequence that does not contain Su(H)-binding site (*ban* con) in the upstream regulatory region of pre-*bantam* are indicated. (Right) Histogram showing enrichment of *ban* genomic sequence surrounding S1, S2, but not the *ban* con region, in the Su(H) ChIP. *E(spl)m8* and *rp49* are positive and negative controls, respectively. ***, *p*<0.0001; *, *p*<0.005, n = 3 repeats. (**G**, **H**) Effects of *ban* LOF and GOF on *E(spl)mγ-GFP* reporter expression in type I NBs located in posterior brain region. **H**, quantification of GFP fluorescence intensity from **G**. *, *p*<0.005; Scale bars: **A**, **B**, 10 μm; **G**, 20 μm.

To better understand how *ban* regulates NB lineage homeostasis, we examined its expression and activity in type II NB lineages, which is the source of the ectopic NBs induced by N hyperactivity. Using a *ban-lacZ* transcriptional reporter, we found that *ban* expression is highly enriched in NBs (Fig 3A and 3C). This is true for both type I and type II NBs. In type II NB lineages, low level of *ban* expression was detected in IPs but not the differentiated neurons. Correlating with this expression pattern, *ban* activity as detected with a GFP sensor, the expression of which correlates inversely with *ban* activity [17], was high in NBs and adjacent IPs but low in differentiated neurons (Fig 3B and 3D). Given that *ban* expression and activity are highly enriched in NBs, we next tested whether this is under N regulation. We found that *ban* expression and activity were elevated by N OE (Fig 3A–3D, S3 Fig), or in α-*adaptin (ada)* mutant condition (S4A and S4B Fig), where N activity is high due to compromised turnover of N receptor on the cell surface [22]; conversely, *ban* expression and activity were diminished when N was knocked down by RNAi (Fig 3A–3D, S3 Fig). These results suggest that *ba*n not only mediates the effect of N on NB lineage homeostasis but its expression and activity are under the control of N. qRT-PCR analysis showed that mature *ban* miRNA level in larval brain was increased by N-OE but decreased by N-RNAi (Fig 3E), similar to the response of known N pathway targets genes (S4C Fig). We next tested whether *ban* is a direct transcriptional target of N signaling. Through chromatin immunoprecipitation (ChIP) using a Su(H) antibody [22], we found that regions of *ban* locus including its promoter region containing putative Su(H) binding sites were preferentially pulled down in the ChIP assay. This is true in both larval brain (Fig 3F) or wing imaginal discs (S4D Fig). qRT-PCR analysis showed that *ban* miRNA level was increased by N-OE but decreased by N-RNAi in the wing discs (S4E Fig) as well. Collectively, these results support that *ban* is a direct transcriptional target of the N pathway.

The differential expression and activity of *ban* in progenitor cells and differentiated daughter cells, and the sharp boundary between cells with high and low *ban* expression and activity, raised the possibility that *ban* may regulate N activity to form a positive feedback loop, a mechanism commonly used to generate “all-or-none” switches during cell fate determination [23]. Using a Notch activity reporter, *E(spl)mγ-GFP* [24], we found that N activity in NBs was increased by *ban* OE, but decreased by *ban-sp* OE (Fig 3G and 3H). This is true for both type II and type I NBs. We chose type I NBs located at stereotypic positions in the posterior brain for analysis because they express the *E(spl)mγ-GFP* reporter at a higher level than other NBs, and their scattered distribution made it easier to do GFP signal quantification. We assume that what is learned from type I NBs on the regulation of N activity by *ban* may well be relevant to type II NBs, but given the differences in Notch function between these cell types, this is an assumption that requires further testing in the future. Our qRT-PCR analysis revealed that other known targets of N were also transcriptionally activated by *ban* OE but repressed by *ban-sp* OE (S4C Fig). These results indicate that *ban* participates in a regulatory network with N to help maintain NSC cell fate.

**Fig 4:**
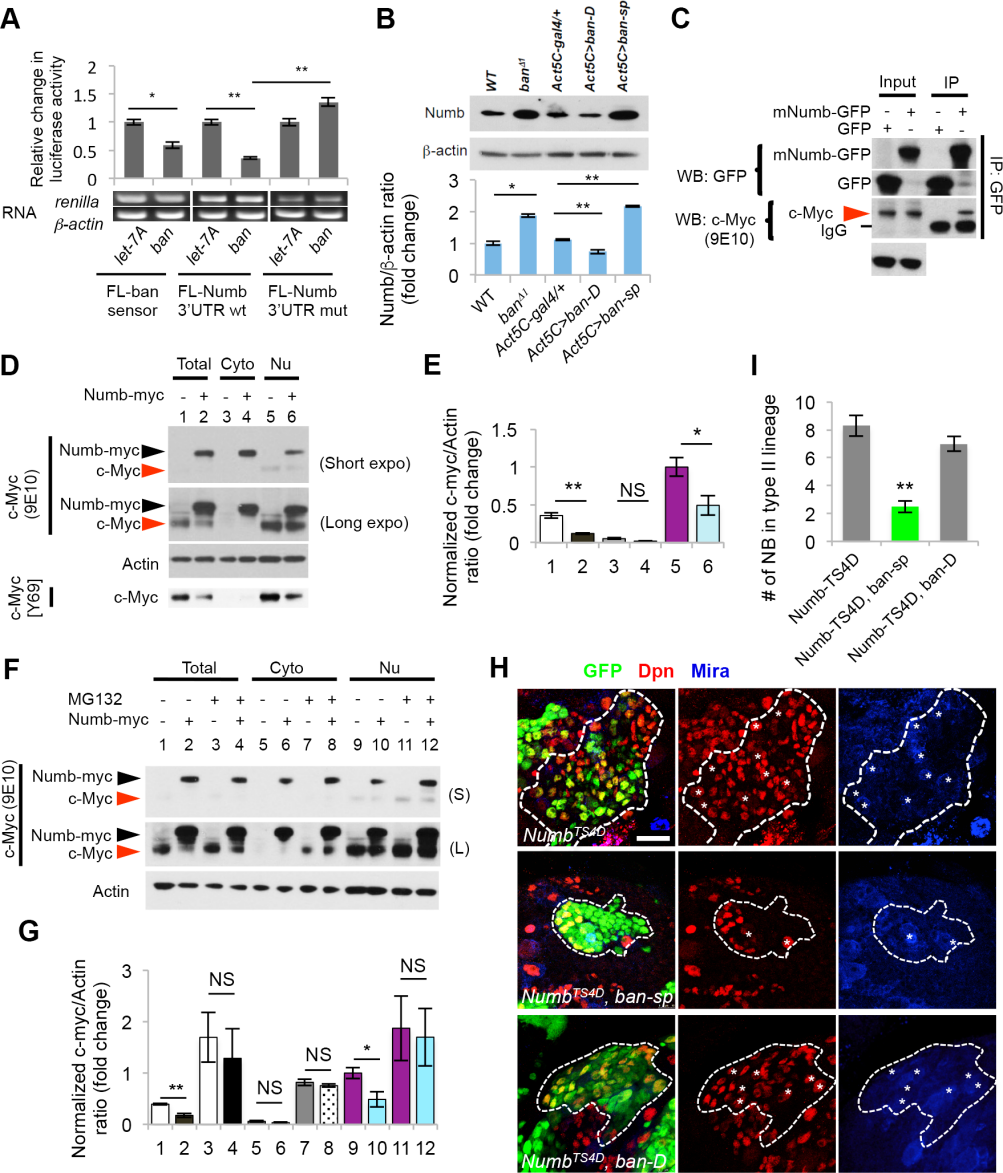
Numb is a target of *ban* and it negatively regulates Myc. (**A**) Translational reporter assay showing targeting of *numb* 3´UTR by *ban* miRNA. Luciferase activity derived from firefly luciferase *(FL)-ban* sensor, renila luciferase (RL)-*numb* 3´UTR, or RL-*numb* 3´UTR^mut^ reporters in response to *ban-5P* miRNA co-expression was normalized with values from *let-7A* control miRNA co-expression. *, *p***<**0.05; **, *p***<**0.001. n = 3 repeats. The gel images under the bar graph represent measurements of *luciferase* mRNA expression by RT-PCR, with *actin* serving as a control. (**B**) Western blot analysis of larval brain extracts showing effects of *ban* LOF or GOF on Numb protein levels. Actin serves as loading control. Bar graph shows quantification of normalized Numb signals from three independent blots. *, *p***<**0.001. n = 3 repeats. (**C**) Co-IP between Numb and c-Myc in HEK293T cells. GFP-mNumb or GFP vector was transfected into HEK293T cells. Anti-GFP immunoprecipitates were probed for GFP and c-Myc by western blot. (**D**) Western blot analysis of c-Myc levels in various cellular fractions from HEK293T cells in response to Numb-myc (myc epitope tagged Numb) expression. The anti-c-Myc (9E10) was used to detect both c-Myc and Numb-myc. c-Myc levels were quantified after normalization to Actin. Total: total lysates (#1, #2); Cyto: cytosol fractions (#3, #4); Nu: nuclear fractions (#5, #6). Effect of Numb on endogenous cMyc level was verified with another anti-c-Myc Ab (Y69). (**E**) Quantification of c-Myc levels from (*D*) after normalization to Actin. c-Myc levels detected by c-Myc (9E10) were quantified in comparison to nuclear lysates in #5. *, *p***<**0.05, **, *p***<**0.005; n **=** 3 independent experiments. (**F**) Effect of the proteasome inhibitor MG132 on endogenous c-Myc level in Numb transfected HEK293 cells. HEK293T cells with or without Numb-myc transfection were incubated in the presence or absence of 10 μM MG132 for 2 hrs. Cell lysates (#1-#12) were analyzed by western blot as indicated. (S): Short exposure; (L): Long exposure. (**G**) Quantification of c-Myc levels upon MG132 treatment shown in **F**. *, *p*<0.05; **, *p***<**0.001; ***, *p***<**0.0001 vs. without MG132 treatment. n **=** 3 independent experiments. (**H**, **I**) Genetic interaction between Numb-TS4D and *ban* in regulating NB homeostasis. Type II NB lineages co-expressing Numb-TS4D and *ban-sp* or Numb-TS4D and *ban-D* are marked with white dashed lines. Asterisks indicate ectopic NBs in the clones. **J**, quantification of NB number in type II NB clones from **I**. **, *p***<**0.00001; n **=** 8–10 NB clones. Scale bars: *H*, 20 μm.

We next sought to identify the target of *ban* that participates in the regulatory network underlying NB fate determination. Although *numb* 3'-UTR has been reported to contain two predicted *ban* binding sites [18], the *ban* binding site(s) responsible for *numb* translational repression remains to be determined. Using the RNAHybrid program [25] for miRNA target prediction, we identified several candidate *ban*-binding sites in *Drosophila numb* mRNA 3´-UTR as well as CDS (S5A Fig). In translational reporter assays, the addition of *numb* 3´-UTR made the translation of the luciferase reporter specifically sensitive to the presence of *ban* but not *let-7* miRNA (Fig 4A). Mutating the seed sequence in a best-predicted *ban*-binding site in *numb* 3´-UTR, which is distinct from the two predicted sites reported previously [18], abolished the sensitivity of the reporter to *ban* (Figs 4A and S5B). These results suggest that *numb* mRNA is a potential target of *ban*. To gather *in vivo* evidence of *ban* regulation of *numb*, we first examined the effect of *ban* LOF and GOF on endogenous Numb expression. Western blot and qRT-PCR analyses showed that in *ban*^*Δ1*^ mutant or when *ban-sp* was ubiquitously expressed, levels of Numb protein (Fig 4B) and mRNA (S5C Fig) expression were significantly increased in the brain, whereas *ban* OE led to a moderate reduction of Numb protein and mRNA levels (Figs 4B and S5C).

We next examined the *in vivo* functional relationship between *ban* and *numb* in NB regulation. Knockdown of *numb* by RNAi in type II NB lineages resulted in enlarged nucleolar size in newly born IPs, and *numb* RNAi rescued the nucleolar size reduction caused by *ban-sp* OE (Fig 2D and 2E). Importantly, the nucleolar size increase caused by *numb* RNAi was Myc-dependent (Fig 2D and 2E; S6A–S6C Fig), as in *ban* OE case (Fig 2A and 2C). These results support the notion that Numb is a key target mediating the effect of *ban* on nucleolar growth.

We further examined the biochemical relationship between Numb and Myc underlying their functional interaction in *ban*-regulated nucleolar growth. In mammalian HEK293 cells, Numb and c-Myc exhibited physical interaction (Fig 4C). Overexpression of Numb led to reduced level of endogenous cMyc (Fig 4D and 4E), an effect abolished by treatment with the proteasome inhibitor MG132 (Fig 4F and 4G), suggesting that Numb affects Myc protein level through the ubiquitin-proteosome system (UPS). We have also examined Myc levels in *Drosophila* larval brain NBs with altered Numb activities. We found that dMyc level is increased when Numb is inhibited by RNAi, and decreased when Numb is overexpressed. This is true for both endogenous dMyc (S7A) or overexpressed dMyc (S7B). We have also tried to examine the effect of altered *ban* activities on dMyc expression. The immunostaining did not reveal consistent clear-cut results as seen in Numb manipulation case, probably because *ban* acts through Numb to indirectly affect dMyc expression, making its effect on dMyc protein level not as robust as Numb. However, when we examined the effect of altered *ban* activity on endogenous dMyc expression by western blot analysis of brain extracts, we saw increased dMyc protein level in *ban* GOF condition and reduced dMyc level in *ban* LOF condition (S7C Fig).

Numb has previously been shown to regulate transcription factor stability by stimulating E3 ligase activity [26]. We found that the effect of Numb in promoting Myc degradation was attenuated by knocking down Huwe1 in HEK293 cells (S7D and S7E Fig). In fly larval brain, Huwe1 RNAi resulted in increased nucleolar sizes of NBs and IPs in type II NB lineages in a Myc-dependent manner (S8A–S8C Fig). Moreover, Huwe1 functionally interacted with Numb and Myc to regulate type II NB maintenance, as indicated by the ability of Huwe1 RNAi to facilitate Myc in rescuing the type II NB loss caused by Numb OE (S8D and S8E Fig).

To examine the effect of *ban* on Numb protein expression specifically in NBs, we first examined Numb protein expression in the NBs in *ban* LOF and GOF FLP-out clones. Our results showed that Numb expression level change in the NBs displays similar trends as detected by the western blot analysis of brain tissues (S9A and S9B Fig), although the difference did not achieve statistical significance. This may be due to the sensitivity of the immunostaining method, the specificity of the antibody, or the relatively high basal expression of Numb in the otherwise wild type NBs. Consistent with the last scenario, when we examined Numb expression in N-V5 overexpression NBs that have lower basal level of Numb, the effect of *ban-sp* in elevating endogenous Numb expression became more significant (S10A and S10B Fig). This result further supports the notion that the translation of *numb* mRNA is regulated by *ban in vivo*.

Previous studies showed that OE of a phospho-mimetic form of Numb (Numb-TS4D) caused ectopic NB formation and tumorous brain growth, an effect likely reflecting a dominant-negative effect of Numb-TS4D in inhibiting endogenous Numb, as co-expression of Numb-WT completely rescued the Numb-TS4D effect [27]. We found that the Numb-TS4D effect was also rescued by *ban-sp* (Fig 4H and 4I), presumably due to elevation of the level of endogenous Numb by *ban-sp* that counteracted Numb-TS4D action. In contrast, co-overexpression of *ban* using *UAS-ban-D* did not change Numb-TS4D effect (Fig 4H and 4I), presumably because endogenous Numb activity has been sufficiently inhibited by Numb-TS4D such that its further translational repression by *ban-D* OE will not have additional phenotypic effect.

**Fig 5:**
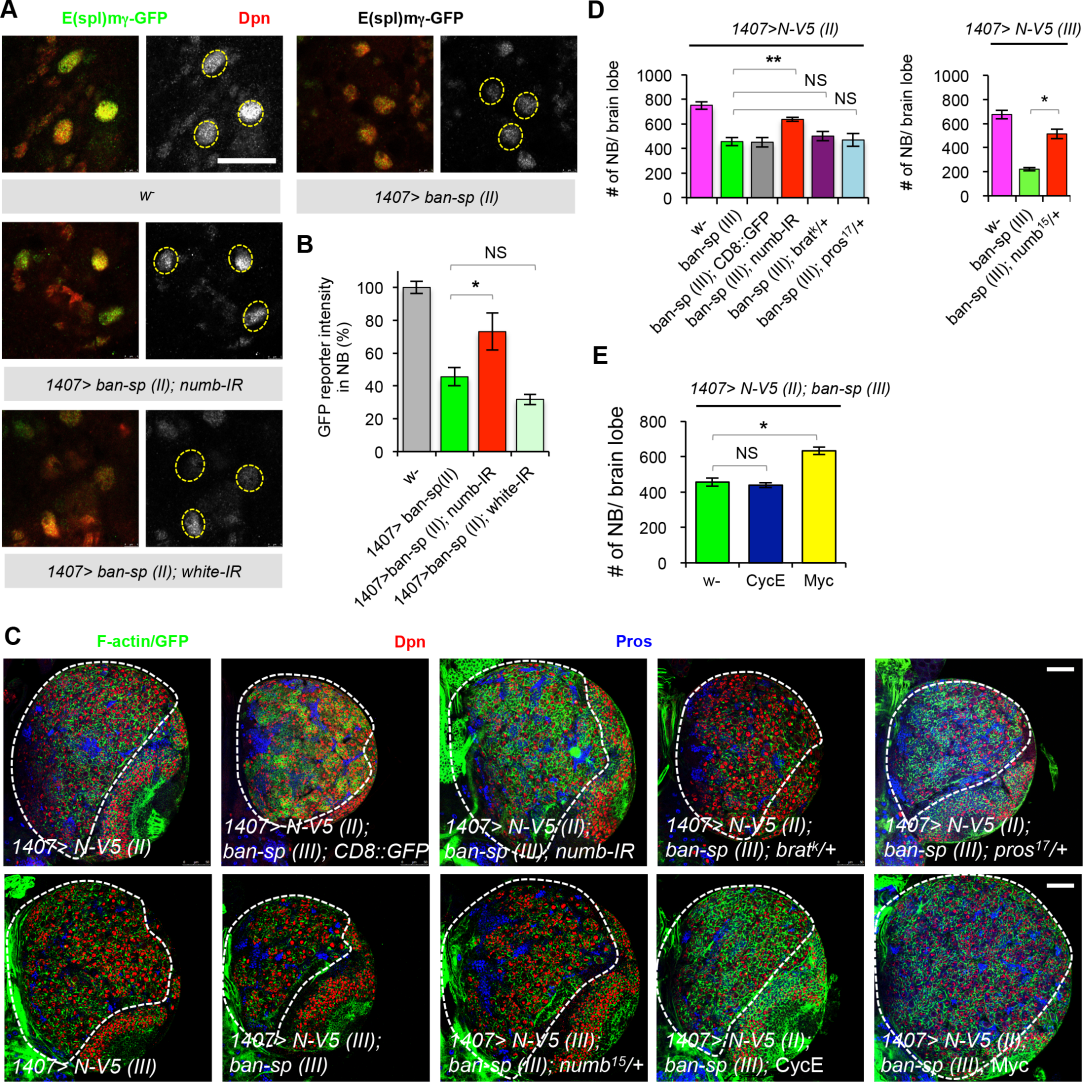
Numb mediates the effects of *ban* in the feedback regulation of N activity and CSC-like NB proliferation. (**A**) Genetic interaction between *ban* and *numb* on the expression of N activity reporter *E(spl)m g-GFP*. The effect of *ban-sp (II)* OE driven by *1407-Gal4* on N activity was rescued by *numb-RNAi*. (**B**) Quantification of data from **A**. *, *p*<0.05 vs. *1407>ban-sp (II)*; n **=** 10–14 type I NBs/genotype. (**C**) The effect of *ban-sp* in blocking N-induced brain tumor formation is sensitive to the gene dosage of *numb* but not *pros* or *brat*. *N-V5 (II)* and *N-V5 (III)* stand for *N-V5* transgenes on the 2nd or 3rd chromosomes, respectively. (**D**) Quantification of data from **C**. **, *p*<0.00001 comparing *1407>ban-sp* (III) with and without *numb-IR* in N-V5 background; *, *p*<0.0005 comparing *1407>ban-sp* (III) with or without removing one copy of *numb*, in N-V5 (II) or N-V5 (III) OE background. n = 5–10 brain samples. (**E**) Myc OE, but not CycE OE, rescued *ban-sp* effect in blocking N-induced brain tumor formation. Data is quantified using images from **C**. *, *p*<0.00001 comparing *1407>N-V5; ban-sp* without Tg expression (w^-^) vs. with Tg expression (Myc or CycE). Scale bars: *A*, 20 μm; *C*, 50 μm.

To further test for a critical role of Numb in mediating the effects of *ban* on N activity and CSC-like growth, we performed genetic epistasis experiments. First, we found that the effect of *ban* inhibition by *ban-sp* in attenuating N activity was mediated by Numb, as *numb* RNAi or removal of one copy of *numb* (Fig 5A and 5B; S11A and S11B Fig) blocked the *ban-sp* effect. In contrast, removal of one copy of *pros* or *brat*, two other genes that were recently identified as *ban* targets that regulate normal NB growth and proliferation [18], was without effect (S11A andS11C–S11E Fig). Consistently, *numb* RNAi or removing one copy of *numb*, but not *pros* or *brat*, effectively rescued the effect of *ban-sp* in attenuating ectopic NB formation and tumor-like growth induced by N hyperactivation (Fig 5C and 5D). In N-OE condition, *numb* RNAi or removing one copy of *numb* alone had no obvious effect on NB number (S12A and S12B Fig). Moreover, promotion of cell growth by Myc-OE, but not cell cycle progression by Cyclin E-OE, which failed to affect nucleolar growth (S12C and S12D Fig), suppressed the effect of *ban-sp* on ectopic NB formation and tumorous growth (Fig 5E). Consistent with Huwe1 being a negative regulator of dMyc, Huwe1 RNAi also suppressed the effect of *ban-sp* on ectopic NB number and tumorous growth (S12E and S12F Fig). Together, these results suggest that at least in N-induced CSC-like NB growth and proliferation, Numb is a key target that mediates the effect of *ban*, and cell growth conferred by the Numb-Myc axis is a key mechanism of NB homeostasis regulation by *ban*.

## Discussion

**Fig 6:**
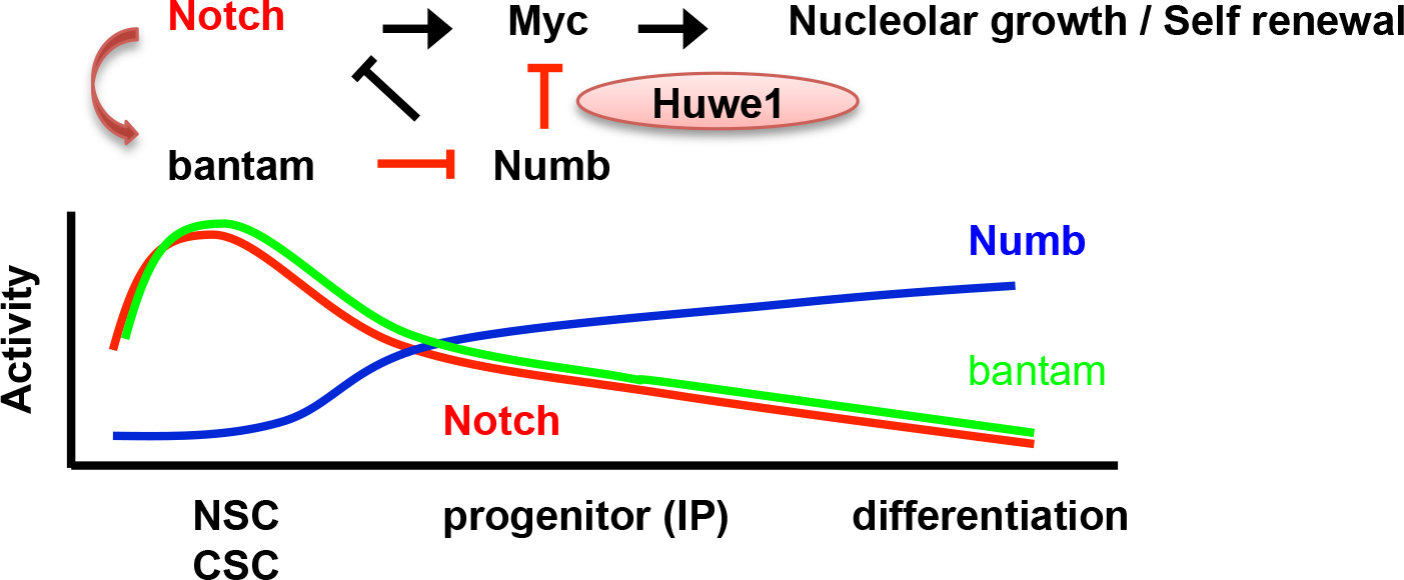
A diagram depicting the regulatory network involving N, *ban*, Numb, and Myc in cell fate determination in NSC/CSC lineages. In stem cells, positive transcriptional regulation of *ban* by N and a feedback regulation of N by *ban* help maintain high N activity and stem cell fate. A key aspect of stem cell maintenance is nucleolar growth promoted by Myc. Myc is a known transcriptional target of N and is independently regulated at the protein stability level by Numb through Huwe1 as shown in this study. As differentiation proceeds, the feedback regulatory loop is weakened, presumably contributed by the asymmetric segregation of Numb, resulting in gradual decline of N and *ban* activities and corresponding increase of Numb activity in differentiated progenies.

By revealing the involvement of the miRNA pathway, here we highlight the complexity of the N signaling network in normal NSCs and tumor-forming CSC-like NSCs. Previous studies implicated critical roles for both canonical and non-canonical N signaling pathways in NSCs and CSC-like NSCs, and revealed particular dependence of CSC-like NB growth on non-canonical N signaling, which involves PINK1, mTORC2, and mitochondrial quality control [9]. Our current study reveals a particular requirement for *ban* in CSC-like NBs induced by N hyperactivation. The CSC-like NB overproliferation induced by hyperactivation of N or N pathway component Dpn (Figs 1A, 1B, 1C–1F, 4I, 4J, 5C and 5D) can all be assumed to be of type II NB origin, since previous studies have clearly established that Notch signaling is essential for the development and/or maintenance of type II NBs, but dispensable for type I NBs, and that hyperactivation of Notch or its downstream effector Dpn induced ectopic CSC-like NB growth by altering the lineage homeostasis of the type II but not type I NBs [3–8, 20, 21]. It would be interesting to test whether, in addition to *ban*’s role in canonical N signaling, there exists a link between *ban* and non-canonical N signaling. Our data indicate that the *ban*-Numb signaling motif regulates NSC/CSC behavior through at least two mechanisms. On one hand, it regulates cell growth and particularly nucleolar growth, through Myc, a known regulator of cellular and nucleolar growth [28]. Consistently, we observed negative regulation of Myc protein level by Numb through Huwe1 and the UPS. c-Myc is an essential regulator of embryonic stem cell (ESC) self-renewal and cellular reprogramming [29], and Myc level and stability can be controlled in stem cells through targeted degradation by the UPS [30, 31], suggesting conserved mechanisms. A key function of the nucleolus is the biogenesis of ribosomes, the cellular machinery for mRNA translation, and previous studies in *Drosophila* have supported the critical role of nucleolar growth in NSC self-renewal and maintenance [7, 32]. On the other hand, the *ban*-Numb axis feedback regulates the activity of N by a double negative regulation, with the end result being positive feedback regulation. This feedback mechanism may help transform initial not so dramatic differences in N activity between NB and its daughter cell generated by the asymmetric segregation of Numb during NB division [33] into “all-or-none” decision of cell fates (Fig 6). Feed-forward regulatory loops, both coherent and incoherent, are frequently found in gene regulatory networks [23], and although *ban* miRNA is not conserved in mammals, miRNAs have been implicated in an incoherent feed-forward loop in the Numb/Notch signaling network in colon CSCs in mammals [34].

Given the role of *ban* in a positive feedback regulation of N and the potency of N hyperactivity in inducing tumorigenesis, one may wonder why *ban* overexpression is not sufficient to cause tumorigenesis. As in any biological systems, feedback regulation is meant to increase the robustness and maintain homeostasis of a pathway. Feedback alone, either negative or positive, should not override the main effect of the signaling pathway. Thus, in the NB system feedback regulation by *ban* is built on top of the available N signaling activity in a given cell and serving to maintain N activity. Because of *ban*’s “fine-tuning” rather than “on/off switching” of Numb expression, its effect on N activity during feedback regulation will also be “fine-tuning”, serving to maintain N activity in NB within a certain range. Overexpression of *ban* in a wild type background may not be sufficient to cause tumorigenesis because N activity is not be elevated to the level sufficient to induce brain tumor as in N-v5 overexpression condition. Consistent with this, the extent of Numb inhibition by *ban* is also modest, not reaching the threshold level of Numb inhibition needed to cause tumorigenesis. Consistent with the notion that feedback regulation by *ban* is built on top of the available N signaling activity in a given cell, and that there is dosage effect of N activity in tumorigenesis, overexpression of *ban* in N-v5 overexpression background further enhanced N-v5 induced tumorigenesis (S1 Fig). It is likely that *ban* or other miRNAs may participate in additional regulatory mechanisms in the N signaling network in *Drosophila*. Of particular interest, it would be interesting to test whether miRNAs may impinge on the asymmetric cell division machinery to influence the symmetric vs. asymmetric division pattern [35], a key mechanism employed by NSCs and transit-amplifying IPs to balance self-renewal with differentiation.

Our results emphasize the critical role of translational control mechanisms in NSCs and CSC-like NSCs. Compared to the heavily studied transcriptional control, our knowledge of the translational control of NSCs and CSCs is rather limited. As fundamental regulators of mRNA translation, miRNAs can interact with both positive and negative regulators of translation to influence gene expression [36, 37]. Thus, miRNA activity can be regulated context-dependently at both the transcriptional and translational levels, which may account for the opposite effect of N on *ban* activity in the fly brain and wing disc [38], although the *ban* genomic locus is bound by Su(H) in both tissues. Whether N regulates the transcription of *ban* or its activity as a translational repressor in the wing disc remains to be tested. With regard to the translation of *numb* mRNA, the conserved RNA-binding protein (RNA-BP) Musashi [39] has been shown to critically regulate the level of Numb protein in mammalian hematopoietic SCs and leukemia SCs [40, 41]. Further investigation into the potential interplay between miRNAs and RNA-BPs in the translational control of Numb in NBs and CSC-like NBs promises to reveal new mechanisms and logic in stem cell homeostasis regulation, with important implications for stem cell biology and cancer biology.

## Materials and methods

### Fly genetics

Fly culture and crosses were performed according to standard procedures and were raised at indicated temperatures. *Drosophila* stocks were obtained from the Bloomington *Drosophila* Stock Center, the Vienna *Drosophila* Resource Center (VDRC), or individual investigators in the *Drosophila* research community. Please see Supplementary Materials and Methods for details.

### Immunostaining and microscopy

For immunostaining of *Drosophila* brains, late third instar larva were dissected, processed for immunohistochemistry, and imaged by confocal microscopy essentially as described [7]. Please see Supplementary Materials and Methods for details of the antibodies used for immunostaining.

### Western blot analysis and quantification

The primary antibodies used for western blot analysis in HEK293T were: chicken anti-GFP (1:20,000; Abcam), mouse anti-c-myc (1:500; 9E10, Santa Cruz Biotechnologies), rabbit anti-c-Myc (1:2000; Y69, Abcam), rabbit anti-c-Myc (1:1000; Cell Signaling Technology), rabbit anti-β-actin (1:20,000; Millipore), mouse anti-Actin (1:20,000; AbD Serotec). Nuclear and cytosolic extracts were obtained using the NE-PER Nuclear and Cytoplasmic Extraction Kit (Thermo Scientific). For western blot analysis of *ban* LOF, GOF, and additional mutants in larval brains, protein extracts were prepared from late third instars of various genotypes, resolved on SDS-PAGE, transferred to Immobilon-P membrane (Millipore) and probed with the indicated antibodies. Please see Supplementary Materials and Methods for details of the other antibodies used for western blotting. Target protein versus loading control band intensities was measured from three independent blots with the Tina2.0 software (raytest Isotopenmessgeraete GmbH, Straubenhardt, Germany) or Image Studio Lite.

### MARCM and flip-out clonal analysis

To generate MARCM clones, larva at 24 h after larval hatching (ALH) were heat-shocked for 1 hr at 38°C and further aged for 72–96 h at 25°C before dissection. For *ban* mutant MARCM clonal analysis, *hsFLP*, *elav-Gal4; UAS-mCD8-GFP; FRT2A*, *tubP-Gal80/TM6b* were crossed to *FRT2A*, *ban*^*Δ1*^ */TM6b* to examine *ban* LOF effects in normal NSCs, or crossed to *N*^*act*^*; FRT2A*, *ban*^*Δ1*^*/TM6b* to examine *ban* LOF effects in CSC-like NBs. For flip-out clonal analysis, *w*, *hsFLP; Actin 5c****>****CD2****>****Gal4*, *UAS-GFP-NLS* was crossed with the indicated *UAS* lines, and 24 h ALH larva were heat-shocked for 1 hr at 38°C and further aged for 72–96 h at 25°C before dissection. Occasionally, two clones may be adjacent to each other, especially in brain tumor backgrounds. These large “fused clones” can be distinguished from true “tumor clones” derived from single CSC-like NBs by the lack of GFP signal at the clone boundary in the former, and the distinct topologies in the organization of the stem cells and differentiated progenies in these two types of clones.

### Data quantification and statistical analysis

For NB cell size or nucleolar size quantification, measurements were performed as previously described [7]. In all Figures, unpaired Student’s *t*-tests were used for statistical analysis between two groups.

## Supporting information

S1 Text Detailed descriptions of supplementary materials and methods, supplementary references, supplementary figure legend, and supplementary tables of PCR primer sequences. (DOCX)

S1 Fig Analysis of the effect of *ban* GOF on CSC preservation. (**A**) Immunostaining of third instar larval brains using the *1407-Gal4*: *Gal80*^*ts*^ system to assess the effect of *ban* GOF (*ban-D* OE) on N-induced NB overproliferation. Green: F-actin; Red: Dpn; Blue: Pros. (**B**) Quantification of data from **A**. **, *p*<0.0001 (*1407-Gal4*: *Gal80*^*ts*^*>N-V5*, *ban-D* vs. *1407-Gal4*: *Gal80*^*ts*^*> N-V5*) in Student’s *t*-test; n = 6–8 brains. Scale bars, 50 μm.
(JPG)

S2 Fig Analysis of the effect of *ban* LOF or GOF on NSC proliferation and growth. (**A**) MARCM analysis of type I and type II NBs in WT or *ban*^*Δ1*^ mutant clones at 120 h ALH. Clones are labeled with GFP in green; type I and II NBs are marked with red and white arrows, respectively. (**B, C**) Quantification of cell sizes of type I (**B**) or type II (**C**) NBs in WT and *ban*^*Δ1*^ mutant clones from **A**. *, *p*<0.005; n = 5–8 clones. (**D**) Quantification of cell size of immature IPs WT or *ban*^*Δ1*^ mutant type II NB clones from **A**. *, *p*<0.005; n = 6 clones. (**E**) Quantification of NB number in WT or *ban*^*Δ1*^ mutant type II NB clones. (NS) Not significant; n = 10 clones. (**F**) Quantification of the number of mature IPs in WT or *ban*^*Δ1*^ mutant type II NB clones from **A**. *, *p*<0.005; n = 6 clones. (**G**) Effects of *ban* GOF (*ban-D* OE) on cell size in type II NBs or immature IPs. The type II NB lineages in late third instar larval brains of WT or *ban-D* OE animals driven by *1407-GAL4* are shown. Green: F-actin; Red: Miranda; Blue: Dpn; Asterisks: type II NBs. (**H**) Quantification of cell sizes of type II NBs in WT and *ban-D* OE brains from **G**. (NS) Not significant; n = 11 brains. (**I**) Quantification of cell sizes of immature IPs in WT and *ban-D* brains from **G**. *, *p*<0.001; n = 6 brains. Scale bars: A, G, 10 μm.
(JPG)

S3 Fig Effects of N OE and N RNAi on *ban* activity and expression in larval brains. (**A**) Posterior and anterior views of *ban-lacZ* transcriptional reporter expression in immunostained WT, N-OE, or N RNAi (N-IR) brains. The *1407-GAL4*, *tub-GAL80*^*ts*^ system was used to induce N-V5 or N-IR transgene expression in both type I and type II NBs. White dashed lines outline the central brains. Zoomed in images and data quantification of *ban* GFP sensor expression in NBs are shown in **Fig 3A and 3C**. (**B**) Posterior and anterior views of *ban* GFP sensor expression in WT, N-OE, or N-IR brains. Zoomed in images and data quantification for LacZ expression in NBs is shown in **Fig 3B and 3D**. Scale bar in **A**, **B**: 50 μm.
(JPG)

S4 Fig Analysis of *ban* transcription in α-*ada* mutant NBs and evidence of feedback regulation of N target gene expression by *ban*. (**A**) Effects of α*-ada* LOF on *ban* activity as monitored with the GFP sensor. GFP sensor of *ban* was undetectable in ectopic type II NBs of α*-ada* homozygous mutants, whereas NBs with N RNAi (N-IR) driven by *1407-GAL4* showed low level *ban* GFP sensor expression. Green: GFP; Red: Dpn; Blue: Pros; Brackets: NBs. Bar graph shows quantification of *ban* sensor GFP fluorescence intensity in NB. *, *p*< 0.001, n = 12 brains. (**B**) Effects of α*-ada* LOF on *ban*-*lacZ* transcriptional reporter expression. Green: F-actin; Red: LacZ, Blue: Pros; Brackets: NBs. IPs in WT or α*-ada* mutant type II NB lineages are indicated with open or closed arrowheads, respectively. Bar graph shows quantification of LacZ immunofluorescence in type II NBs. *, *P*< 0.001, n = 12 brains. (**C**) ChIP analysis testing Su(H) binding to *ban* genomic DNA in wing discs. Quantitative PCR analysis revealed enrichment of *ban* sequences surrounding two putative Su(H)-binding sites (S1 and S2), but not a control *ban* sequence that does not contain a predicted Su(H)-binding site (*ban* con). See Fig 2F for positions of *ban* S1, S2, and con in the *ban* locus. *E(spl)m8* and *rp49* are positive and negative controls, respectively. **, *p*< 0.001, *, *p*< 0.05, n = 3 brains. (**D**) Quantitative RT-PCR analysis of mRNA levels of Notch target genes *E(spl)m3*, *E(spl)m7* and *E(spl)mγ* in larval brains overexpressing *ban* (*1407>ban-D*) or *ban-sp* (*1407>ban-sp*). Data show the mean of 3 independent experiments after normalization with *rp49*. Error bars indicate s.e.m. *, *p*< 0.05. (**E**) Quantitative RT-PCR analysis of *ban* levels in wing discs. *ban* levels were normalized to 2S rRNA. *, *p*<0.01, n = 3 repeats. Scale bar: **A**, **B**, 10 μm.
(JPG)

S5 Fig Predicted *ban-5p* and *ban-3p* target sites in *numb* transcript. (**A**) Schematic of *numb* locus showing genomic organization and predicted target sites for *ban-5p* and *ban-3p*. ORF is indicated in black and UTR sequences in white. Sequences of predicted base-pairings between *ban-5p* or *ban-3p* and *numb* mRNA were identified in ORF and 3'UTR using the RNAHybrid program available at [http://bibiserv.techfak.uni-bielefeld.de/rnahybrid/submission.html]. Numbering is relative to first nucleotide of *numb* 3’UTR or ORF. Free energies for binding between *ban* miRNA and each target sites are listed. (**B**) Mutagenesis of *numb* ‘UTR for luciferase reporter assay. Left: The red arrow indicates the location of one predicted *ban-5P* target site in *numb* 3'UTR (position at 82) used for mutagenesis to generate *RL*-*numb* 3'UTR^mut^ construct in **Fig 4A**. Right: Red labeled nucleotides indicate mutations introduced in the *RL*-*numb* 3'UTR^mut^ construct. (**C**) Quantitative RT-PCR analysis of *numb* mRNA levels in *ban* (*Act>ban-D*) or *ban-sp* (*Act>ban-sp*) overexpressing larvae. The qRT-PCR analysis is correlated with the data shown in **Fig 4B**. Data show the mean of 3 independent experiments after normalization with *rp49*. Error bars indicate s.e.m. *, *p*< 0.05.
(JPG)

S6 Fig Analysis of the effect of *numb* RNAi and *myc* RNAi on cell fate determination in type II NB lineages. (**A**) The reduction of IP number and type II NB lineage size from *myc* RNAi, but no loss or cell fate transformation from *numb* RNAi driven by *1407-Gal4*. At 120 h ALH, the type II NB lineages in larval central brains were analyzed. *numb* and *myc* double RNAi behaves similar phenotypes as *myc* RNAi. Type II NB lineages are marked with white dashed lines. Asterisks: type II NBs; open arrowheads: immature IPs; white arrowheads: mature IPs. Green: F-actin; Red: Ase; Blue: Dpn. (**B**) Quantification of number of immature IP from **A**. *, *p*<0.05; **, *p***<**0.01; ***, *p***<**0.001. n = 7 brains. (**C**) Quantification of number of mature IP from **A**. *, *p*<0.05, n = 7 brains. Scale bars: A, 10 μm.
(JPG)

S7 Fig Evidence for the involvement of Huwe1 in the regulation of Myc protein level by Numb. (**A**) Effects of Numb RNAi and Numb OE on Myc protein expression. Top panels: Posterior surface views of whole brains are shown. *1407/+*, *1407>numb-IR*, *1407>Numb*, and *1407>myc-IR* were immunostained at 120 h ALH for dMyc. White dotted line indicates the boundary between optical lobe (OL) and central brain (CB) regions. Bottom panels: zoomed in images of dMyc staining in type I NBs in top panels. Note that dMyc expression in the nucleus is abolished in *1407>myc-IR* brain. Green: Miranda; Red: dMyc. (**B**) Supporting evidence that Numb OE attenuates Myc protein expression. Top panels: Whole brains of *1407> dMyc* and *1407> dMyc; Numb* immunostained at 120 h ALH for dMyc. Type II NB lineages are outlined. Bottom panels: zoom in images of Myc staining in NBs. Note that *UAS-Numb* transgene expression driven by *1407-GAL4* resulted in loss of type II NB lineages. Green: Miranda; Red: dMyc. (**C**) Western blot analysis of larval brain extracts showing effects of *ban* LOF or GOF on Numb protein levels. Actin serves as loading control. (**D**) Western blot analysis assessing the effect of Huwe1 RNAi on c-Myc protein level reduction caused by Numb overexpression in HEK293T cells. Cells with or without myc-tagged Numb (Numb-myc) expression and co-transfected with Huwe1 siRNA or control siRNA were fractionated and subjected to western blot analysis with the indicated antibodies. Efficient knockdown of Huwe1 by siRNA was revealed by anti-Huwe1 western blot. Total: total lysate; Cyto: cytosol fraction; Nu: nuclear fraction. (**E**) Quantification of normalized nuclear c-Myc levels in cells expressing Numb-myc vs. cells not expressing Numb-myc from **E** by comparing c-Myc levels after normalization with Actin. *, *p*<0.01. n = 3 independent experiments. Scale bars: **A**, **C**, 50 μm (top panels) and 20 μm (bottom panels).
(JPG)

S8 Fig Numb and Huwe1 regulate Myc protein level and function. (**A**-**C**) Genetic interaction between *huwe1* and *myc* in nucleolar size regulation. Larval brain expression of transgenes was driven by *1407-Gal4*. Green, Fibrillarin; Red, Miranda. Brackets: type II NBs. Nucleoli of immature IPs are indicated by arrowheads. (**B**) Quantification of nucleolar size in type II NBs from **A**. **, *p*< 0.0001; n = 7 brains. (**C**) Quantification of nucleolar size in immature IP of type II NB lineages from **A**. *, *p*< 0.01; **, *p*< 0.0001; n = 7 brains. (**D**) Huwe1 RNAi promotes the ability of Myc OE to rescue the type II NB loss caused by Numb OE. Single optical section of type II NB lineages in WT, *1407>Numb*, *1407>Numb; dMyc*, *1407>Numb*, *huwe1 RNAi; dMyc*, and *1407> huwe1 RNAi* larval brains marked with white dashed lines were immunostained for NBs (Dpn), differentiated cells (Pros), and cell cortex (F-actin). Each type II NB expressing Dpn^+^ is indicated by a star. Yellow dotted line indicates the boundary between the optical lobe (left) and the central brain (right) region. Co-expression of Dicer2 was applied in all genotypes to enhance RNAi effect. (**E**) Quantification of number of type II NBs from **D**. **, *p*< 0.01 (vs Numb; dMyc); n = 8 brains. Scale bars: **A**, 10 μm; **D**, 50 μm.
(JPG)

S9 Fig Analysis of the effect of *ban* GOF or LOF on Numb expression. (**A**) Immunostaining of Numb expression in flip-out *ban* GOF (*ban-D*) and *ban* LOF (*ban-sp*) clones in larval brains. Clones and non-clones in the same brains were distinguished by the expression of the GFP marker and the type I NB lineages are outlined with yellow dashed line. Green: GFP; Red: Numb; Blue: pH3; Yellow arrowheads: Numb expression located at the basal side of NB cortex. (**B**) Quantification of Numb expression of type I NBs from **A**. n = 5–8 brains. Scale bars, 10 μm.
(JPG)

S10 Fig Effect of *ban* inhibition by *ban-sp* on Numb protein level in N-OE larval brain NBs. (**A**) Immunostaining of Numb protein in N-V5 (*1407-Gal4*:*Gal80*^*ts*^*>N-V5*) larval brains with or without *ban-sp* co-expression. Arrows indicate dividing NBs stained for α-Tubulin in blue and asymmetrically localized crescent-shaped Numb protein in red. (**B**) Quantification of the fluorescence intensity of Numb protein in dividing NBs from **A**. The analysis was done by measuring Numb protein immunofluorescence in the area with asymmetrically localized Numb protein crescent and normalize that with α-Tubulin levels. *, *p*<0.01, n = 5 brains. Scale bar: **A**, 10 μm.
(JPG)

S11 Fig Evidence that Numb mediates the effect of *ban* in the feedback regulation of Notch activity. (**A**) The effects of *pros*, *numb*, or *brat* gene dosage on *ban-sp* induced reduction of *E(spl)mγ-GFP* reporter expression. Yellow dashed circle marks type I NBs located in the posterior brain with *E(spl)mγ-GFP* reporter expression. (**B**-**E**) Quantification of the fluorescence intensity of *E(spl)mγ-GFP* reporter in type I NBs from **A**. Expression of the reporter is not altered by *numb* RNAi or loss of one copy of *numb* as in *numb*^*15*/+^ condition in an otherwise wild type background. (**B**) In *ban-sp* overexpressing larval brain, *E(spl)mγ-GFP* reporter expression is sensitive to the loss of one copy of *numb*, but not *brat* (**D**) or *pros* (**C**). *ban-sp* (II) and *ban-sp* (III) in **A** indicate *ban-sp* transgenes located on the II or III chromosomes. **, *p*<0.0001 comparing *1407>ban-sp (III)* with or without loss of one copy of *numb*. n = 6–12 brains in **B**-**D**. (**E**) Loss of one copy of *pros* or *brat* has no effect on *E(spl)mγ-GFP* reporter expression in type I NBs. n = 6 brains. Scale bars: **A**, 20 μm.
(JPG)

S12 Fig Evidence for the critical involvement of cell growth but not cell cycle progression in *ban*-mediated ectopic CSC formation induced by N. (**A**) *numb* RNAi or heterozygosity alone has no effect on N*-*induced brain tumor growth. Larval brains at 120h AHL were stained for Dpn (NBs), Pros (differentiated cells), and F-actin (cell cortex). (**B**) Quantification of total number of NBs is shown in **A**. (**C**) Overexpression of the cell cycle regulator CycE did not alter the effect of *ban-sp* overexpression in reducing nucleolar size of IPs in type II NBs. Green: Fibrillarin; Red: CycE; Blue: F-actin; Brackets: NBs. Arrowheads: nucleoli of immature IPs. (**D**) Quantification of nucleolar size of immature IPs in type II NB lineages from **C**. Transgenes were driven by the NB-specific *1407-Gal4*. *, *p*< 0.005. n = 8 brains. (**E**) Effect of Huwe1 inhibition by RNAi on the phenotypes caused by *ban-sp* overexpression in N*-*induced brain tumor growth. Green: F-actin or GFP; Red: Dpn, Blue: Pros. (**F**) Quantification of the number of NBs shown in **E**. *N-V5* (II) and *ban-sp* (III) in **E** indicate transgenes located on the II or III chromosomes. *, *p*< 0.005. n = 8 brains. Scale bars: **A**, **E**, 50 μm; **C**, 5 μm.
(JPG)

S1 Table PCR primers used in ChIP analysis. (DOCX)

S2 Table Primers used in quantitative RT-PCR analysis. (DOCX)
