# Supplementary material for: The bantam microRNA acts through Numb to exert cell growth control and feedback regulation of Notch in tumor-forming stem cells in the Drosophila brain
Source: PLoS Genet. 2017 May 17;13(5):e1006785. doi: 10.1371/journal.pgen.1006785 (PMC5453605; doi:10.1371/journal.pgen.1006785)
Supplement: S1 Text — (DOCX) [file pgen.1006785.s001.docx]

**SUPPLEMENTARY MATERIAL**

**SUPPLEMENTARY METHODS**

**Fly Genetics**

Fly culture and crosses were performed according to standard procedures and raised at indicated temperatures. *Drosophila* stocks used in this study are: *ban^∆1^,* *ban GFP* sensor, *ban-lacZ,* and *UAS-ban-D* [1], *UAS-ban-sp* (on II and III) [2], *1407-Gal4* [3], *UAS-N-V5* (on II and III) [4] , *UAS-Notch^act^* (*P{w[+mC] = UAS-Dl∷N.ΔECN}B2a2*) (BL5830), *UAS-dpn* [5] , *E(spl)mγ-GFP* [6], *UAS-Numb-GFP*, *UAS-Numb* [7], *UAS-Numb-TS4D* [8], *UAS-dmyc* [9], *UAS-CycE* (BL4781), *UAS-numb-IR* (BL35045), *UAS-notch-IR* (v1112, v27229; VDRC), *brat^k06028^*  [10], *pros^17^* [11], *UAS-myc-IR* (v106066, v17487, VDRC), *numb^15^* [12], *Dcr-1^Q1948X^* (BL32067), *white-RNAi* (BL35573), *UAS-Dicer2* (v60008, VDRC), *{PZ} α****-*** *Adaptin^06694^* (*α-ada*) (BL12319) [13], *UAS-huwe1-IR* (BL36715, BL36714; v26937, VDRC), *MS1096-Gal4* (BL8860) [14], *UAS-mCD8::GFP* (BL5130). BL stocks were obtained from Bloomington *Drosophila* Stock Center (USA). For N knockdown by RNAi, *Dicer2* was co-expressed to achieve efficient RNAi effects as previously described [15].

**Chromatin Immunoprecipitation (ChIP)**

ChIP assay was performed based on previously described protocols [15]. *W^1118^* 3^rd^ instar larval brains or wing imaginal discs were disscted and fixed in 1% formaldehyde for 20 min as described, and cross-linking reaction was stopped by adding 0.125M glycine solution. To obtain nuclear extract, 10% NP40 was added (resulting in 0.5% final conc.) and samples were vortexed for 10 seconds, followed by spinning for 5 min at 5000rpm. After three times of washing, pellets were resuspended in ChIP buffer [20 mM Tris-HCl (pH 8.0), 150 mM NaCl, 2 mM EDTA, 1% TritonX-100, 0.01% SDS, protease inhibitor] plus 1% SDS, and sonicated with 20 cycles of 10 second on and 20 second off and using 30% Amplitute (Vibra Cell, USA). Following centrifugation (13,000*g* for 15 min at 4°C), supernatant containing sheared chromatin were examined by DNA gel to confirm average DNA length of 0.3-0.5 kb. As input sample, 10% supernatant was set aside. For each ChIP assay, 2 μL of anti-Su(H) (Santa Cruz Biotechnologies, sc-15813) or goat polyclonal IgG control (Santa Cruz Biotechnologies, sc-2028) were added and samples were further incubated with Magna ChIP™ Protein G Magnetic Beads (Millipore, USA) overnight at 4°C. The beads were washed once in ChIP buffer, twice in wash buffer (20 mM Tris-HCl (pH 8.0), 50 mM NaCl, 2 mM EDTA, 1% Triton X-100, 0.1% SDS), and once in final wash buffer (10 mM Tris-HCl (pH 8.0), 250 mM LiCl, 1 mM EDTA, 1% NP-40, 1% SDS). After reversing the cross-links by incubating inputs as well as immunoprecipitated chromatins at 65 degrees, DNA samples were purified by using ChIP DNA Clean and Concentrator^TM^ kit (Zymo Research). Real-time PCR experiments were performed as described (Song and Lu, 2011), using primers designed against DNA sequences 150-250 bp upstream and downstream of the Su(H) binding sites and E box motif. Results were quantified using the Percent Input method. Primer sequences are listed in Supplementary Table 1.

**Immunohistochemistry**

For *Drosophila* brain immunostaining, larvae were dissected in Schneider’s medium (Invitrogen) or PBS and fixed with 4% formaldehyde in PEM buffer (100 mM PIPES at pH 6.9, 1 mM EGTA, 1 mM MgCl_2_) for 21 min at room temperature. Larval brains were imaged by confocal microscopy as described [15]. Antibodies used for immunostaining included: chicken anti-β-gal (1:2000; Abcam), chicken anti-GFP (1:2000; Abcam), rabbit anti-Dpn (1:1000; [16]), guinea pig anti-Dpn (1:1000; J. Skeath, [17]), mouse anti-Pros (1:100, Developmental Studies Hybridoma Bank [DSHB]), guinea pig anti-Numb (1:1000; [17]), mouse anti-fibrillarin (1:20; Abcam), rat anti-α-Tubulin (1:1000, AbD Serotec), mouse anti-Myc (1:50, [18]; and 1:2 for P4C4B10, [19]), rabbit anti-CycE (1:1000; Santa Cruz Biotechnology, sc-33748), rabbit anti-Ase (1:400; unpublished, Y. Song), rabbit anti-Miranda (1:1000; [20]), rabbit anti-p-Histone3 (1:1000; Millipore). Corresponding secondary antibodies from Molecular Probe (1:200 dilution) were incubated for overnight at 4 degrees. Samples were mounted with vectashield mounting medium (Vector Labortaries).

**Inducible Gene Expression System**

Embryos of the control WT, *1407-GAL4: tub-GAL80^ts^>UAS-N-V5*, *1407-GAL4: tub-GAL80^ts^ >UAS-N-IR/UAS-Dicer2*, and *1407-GAL4: tub-GAL80^ts^>UAS-N-V5/UAS-ban-sp* genotypes were collected for 24 hr after egg laying and allowed to develop at 22**°**C (permissive temperature). Larva at 24 h after larval hatching (ALH) were shifted to 29**°**C (restrictive temperature) until aged at 120 h ALH and processed for dissection and immunostaining.

**Luciferase Assays**

The cloning of *Drosophila* *numb* 3′UTRs was performed by RT-PCR on total RNAs. The primers for amplifying were, 5′ primer: 5′ GCTCTAGACAGGGGCAGGATCGGGCCA AACA**GAGAATCG**ACAGGGGCAGGATCGGGCCAAACA**GAGAATCG**AAGATCTTC-3′; 3′ primer: 5′- GAAGATCTTCGATTCTCTGTTTGGCCCGATCCTGCCCCTG TCGATTCTCTGTTTGGCCCGATCCTGCCCCTGTCTAGAGC-3′, and 5′ primer: 5′ G AAGATCTCAGGGGCAGGATCGGGCCAAACA**GAGAATCG**ACAGGGGCAGGATCGGGCCAAACA**GAGAATCG**AGCGGCCGCTAAACTAT; 3′ primer: 5′- ATAGT TTAGCGGCCGCTCGATTCTCTGTTTGGCCCGATCCTGCCCCTGTCGATTCTCTGTTTGGCCCGATCCTGCCCCTGAGATCTTC. Target sequences were inserted into cloning sites of *pRL-TKlet7A* (Addgene), using the *Xba*I and *NotI* restriction sites. The miRNA-binding site mutation in *numb* 3'UTR was shown in Figure S5, which was generated by PCR using primers with the indicated nucleotide changes. For *ban* sensor luciferase analysis, the sensor plasmid was obtained from Dr. S. Cohen, and subcloned into *pcDNA3-*firefly luciferase (gift from Dr. Phyllis A. Dennery). Transfection and luciferase reporter assays were performed in HEK293Tcells as described previously [21].

**Quantitative RT-PCR (qRT-PCR)**

Over 20 larval brains from synchronized third instar larva (54–58 h ALH) were dissected in Schneider’s media. Total RNA was extracted using the easy-BLUE (TM) reagent (iNtRON biotechnology). All RNA samples were treated with RNase-free DNase I (Promega). cDNA was synthesized using a SuperScript III First-Strand Synthesis System (Invitrogen). For quantitative RT-PCR analysis, ABI Prism 7900 Sequence Detection System (Applied Biosystems) and SyberGreen PCR Core reagents (Applied Biosystems) were used. mRNA level was expressed as the relative fold change against the normalized *rp49* mRNA. The comparative cycle threshold (Ct) method (User Bulletin 2, Applied Biosystems) was used to analyze the data. Primer sequences used in the analysis are listed in Supplementary Table 2.

**Quantification of *bantam* miRNA and miRNA TaqMan Assay**

Two rounds of RNA collection were performed for enrichment of small RNA in quantification of *bantam* miRNA. First, twenty larval brains or wings were homogenized and total RNAs were isolated by Acid-Phenol:Chloroform organic extraction. Small RNA enriched samples were collected on a glass-fiber filter using the *mir*Vana miRNA isolation kit (Ambion) according to manufacturer's instruction. Small RNA enriched samples (10 ng each) were used in reverse transcription reactions with the TaqMan MicroRNA Reverse Transcription Kit (ABI by Life Technologies). Quantitative PCR was performed using the Taqman Universal PCR Mastermix (Life Technologies) and custom Taqman primers for mature *bantam-5p* miRNA and 2S rRNA (ABI by Life Technology). Measurements of *bantam* mature transcript level were normalized to 2S-rRNA.

**Plasmid and siRNA Transfection in HEK293T Cells**

For expression of Numb-myc [, the plasmid was transfected into HEK293T cells, and cell lysates were prepared in lysis buffer with 50 mM Tris (pH 7.5), 150 mM NaCl, 1mM EDTA, 1% Trinton X-100 containing protease inhibitor cocktails (Biotool) plus phosphatase inhibitors (Biotool) at 24 hr posttransfection. To knockdown Huwe1 in normal or Numb-myc expressing cells, cells plated for 1 day were first transfected with siRNAs. After 24 hr incubation with siRNAs, cells were subject to second transfection with Numb-myc. The transfection of plasmids and Stealth siRNA (Invitrogen) were performed with Fugene 6 (Promega) and Lipofectamine RNAi MAX (Invitrogen), respectively, according to manufacturer's instructions. Validated siRNAs for Huwe1 (HSS145435 and HSS145436) and a control siRNA (GC Duplex, 462001) were obtained from Invitrogen. For proteasome inhibition, transfected cells were treated with 10 μM MG132 at 37 degrees for 2 hr preceding cell lysate preparation.

**Immunoprecipitation and Western Blot Analysis**

For immunoprecipitation, transfected HEK293T cells with pEGFPC1-mouse Numb (mNumb) or pEGFPC1 plasmid were lysed in cold lysis buffer, followed by immunoprecipitating using anti-GFP magnetic beads (Medical & Biological Laboratories) at 4°C overnight. The immunoprecipitates were washed four times with lysis buffer and incubated at 95°C for 5 min in SDS sample buffer. Samples were subjected to SDS-PAGE followed by western blotting with indicated antibodies. The primary antibodies used for western blot analysis in supplementary figures were: rabbit anti-Huwe1 (1:1,000; LifeSpan BioSciences, Inc.), mouse anti-c-myc (1:500; 9E10, Santa Cruz Biotechnologies), rabbit anti-c-Myc (1:2000; [Y69], Abcam), mouse anti-Actin (1:20,000; AbD Serotec).

**Image Analysis and Quantification**

Images were taken with the Leica TCS SP5 AOBS confocal microscope. For quantifying the levels of Numb in dividing NBs, single confocal sections of the larval brains were obtained using identical gain/offset settings. Mean fluorescence intensity of the signal was measured using LAS AF (Leica) measuring tool for Numb expression along the basal side of the NB cortex, when the measured NBs appearing to be forming Numb cortical crescents at similar mitotic stages of the cell cycle. Measurements of *E(spl)mγ****-****GFP*, *ban* sensor GFP, and *ban*-lacZ were performed similarly, using identical settings for image collection and then by circling NB for their GFP or LacZ intensity quantification. For NB number analysis, counting analysis was performed with Adobe Photoshop tool. Histograms were generated using either Microsoft Excel or GraphPad Prism software.

**Statistical Analysis**

All *p* Values were calculated using unpaired Student's *t* test. For the quantification in all figures, the mean **±**S.D is shown unless indicated otherwise.

**SUPPLEMENTARY REFERENCES**

1. Brennecke J, Hipfner DR, Stark A, Russell RB, Cohen SM (2002). bantam encodes a developmentally regulated microRNA that controls cell proliferation and regulates the proapoptotic gene hid in Drosophila. *Cell* 113, 25-36.

2. Becam, I., Rafel, N., Hong, X., Cohen, S.M., Milán, M. (2011). Notch-mediated repression of bantam miRNA contributes to boundary formation in the Drosophila wing. *[Development](http://dx.doi.org/10.1242/dev.064774)* [138, 3781-3789.](http://dx.doi.org/10.1242/dev.064774)

3. Luo, L., Liao, Y.J., Jan, L.Y., and Jan, YN (1994). Distinct morphogenetic functions of similar small GTPases: Drosophila Drac1 is involved in axonal outgrowth and myoblast fusion. *Genes Dev* 8, 1787–1802

4. Kanwar and Fortini (2008). The big brain aquaporin is required for endosome maturation and notch receptor trafficking. *Cell* 133, 852--863

5. San-Juan B. P., Baonza A. (2011). The bHLH factor deadpan is a direct target of Notch signaling and regulates neuroblast self-renewal in Drosophila. Dev Biol 352, 70-82.

6. Almeida MS, Bray SJ (2005). Regulation of post-embryonic neuroblasts by Drosophila Grainyhead. *Mech Dev* 122, 1282-93.

7. Knoblich, J.A., Jan, L.Y., Jan, Y.N (1997). Asymmetric segregation of the *Drosophila* numb protein during mitosis: Facts and speculations. *Cold Spring Harb Symp Quant Biol* 62:71–77

8. Ouyang Y, Petritsch C, Wen H, Jan L, Jan YN, Lu B (2011). Dronc caspase exerts a non-apoptotic function to restrain phospho-Numb-induced ectopic neuroblast formation in Drosophila. *Development* 138, 2185-2196.

9. Zaffran S, Chartier A, Gallant P, Astier M, Arquier N, Doherty D, Gratecos D, Semeriva M. (1998). A *Drosophila* RNA helicase gene, pitchoune, is required for cell growth and proliferation and is a potential target of d-Myc. *Development* 125, 3571–3584

10. Arama, E, Dickman D, Kimchie Z, Sheam A, Lev, Z. (2000). Mutations in the beta-propeller domain of the Drosophila brain tumor (brat) protein induce neoplasm in the larval brain. *Oncogene* 19, 3706-3716.

11. Doe, C. Q., Chu-LaGraff, Q., Wright, D. M. and Scott, M. P. (1991). The prospero gene specifies cell fates in the *Drosophila* central nervous system. *Cell* 65, 451-464.

12. Bhalerao S, Berdnik D, Torok T, Knoblich JA (2005). Localization-dependent and -independent roles of numb contribute to cell-fate specification in Drosophila. *Curr Biol* 15, 1583–1590.

13. Gonzalez-Gaitan, M., and Jackle, H. (1997). Role of Drosophila alpha-adaptin in presynaptic vesicle recycling. *Cell* 88, 767–776.

14. Capdevila J., Guerrero I. (1994). Targeted expression of the signaling molecule decapentaplegic induces pattern duplications and growth alterations in *Drosophila* wings. *EMBO J* 13, 4459–4468.

15. Song, Y., and Lu, B. (2011). Regulation of cell growth by Notch signaling and its differential

requirement in normal vs. tumor-forming stem cells in Drosophila. *Genes Dev* 25: 2644-2658.

16. Bier, E., Vaessin, H., Younger-Shepherd, S., Jan, L. Y. and Jan, Y. N. (1992) deadpan, an essential pan-neural gene in Drosophila, encodes a helix-loop-helix protein similar to the hairy gene product. *Genes Dev* 6, 2137 -2151

17. O'Connor-Giles K. M., Skeath J. (2003). Numb inhibits membrane localization of Sanpodo, a four-pass transmembrane protein, to promote asymmetric divisions in *Drosophila*. *Dev. Cell* **5**, 231-243

18. Galletti M et al. (2009) Identification of domains responsible for ubiquitin-dependent degradation of dMyc by glycogen synthase kinase 3beta and casein kinase 1 kinases. *Mol Cell Biol* 29, 3424-3434.

19. Prober, D. A. and Edgar, B. A. (2000). Ras1 promotes cellular growth in the Drosophila wing. *Cell* 100, 435 - 446.

20. Ikeshima-Kataoka., et al. (1997). Miranda directs Prospero to a daughter cell during Drosophila asymmetric divisions. *Nature* 390, 625–629.

21. Gehrke, S. et al. (2010). Pathogenic LRRK2 negatively regulates microRNA-mediated translational repression. *Nature* 466, 637–641.
