# Supplementary figures and images for: The bantam microRNA acts through Numb to exert cell growth control and feedback regulation of Notch in tumor-forming stem cells in the Drosophila brain
Source: PLoS Genet. 2017 May 17;13(5):e1006785. doi: 10.1371/journal.pgen.1006785 (PMC5453605; doi:10.1371/journal.pgen.1006785)

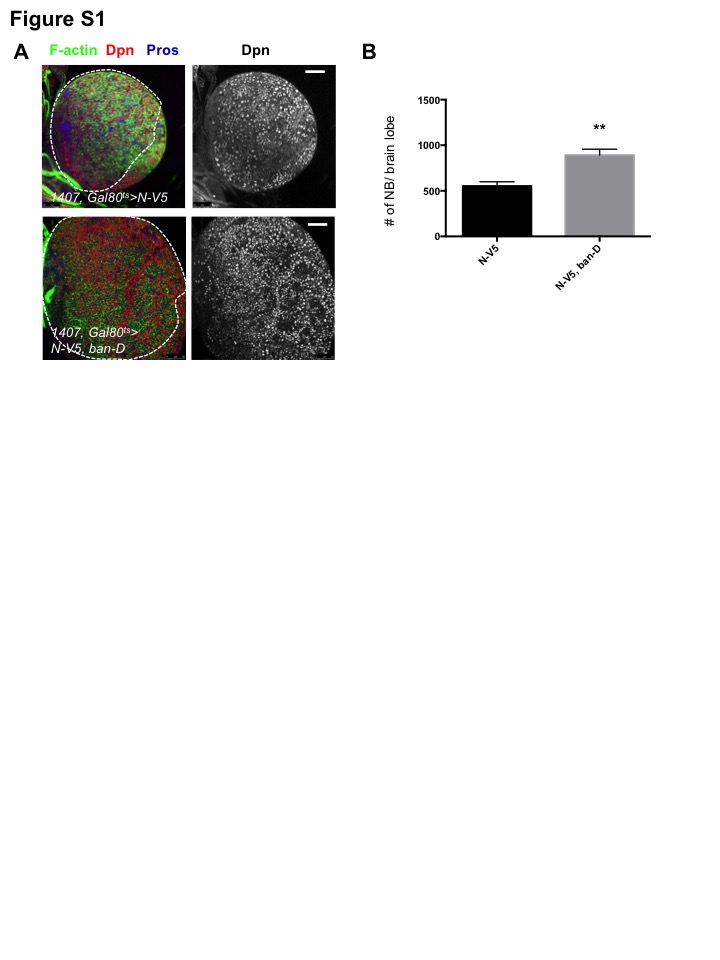

Supplement: S1 Fig — (A) Immunostaining of third instar larval brains using the 1407-Gal4: Gal80ts system to assess the effect of ban GOF (ban-D OE) on N-induced NB overproliferation. Green: F-actin; Red: Dpn; Blue: Pros. (B) Quantification of data from A. **, p<0.0001 (1407-Gal4: Gal80ts>N-V5, ban-D vs. 1407-Gal4: Gal80ts> N-V5) in Student’s t-test; n = 6–8 brains. Scale bars, 50 μm. (JPG) [file pgen.1006785.s002.jpg]

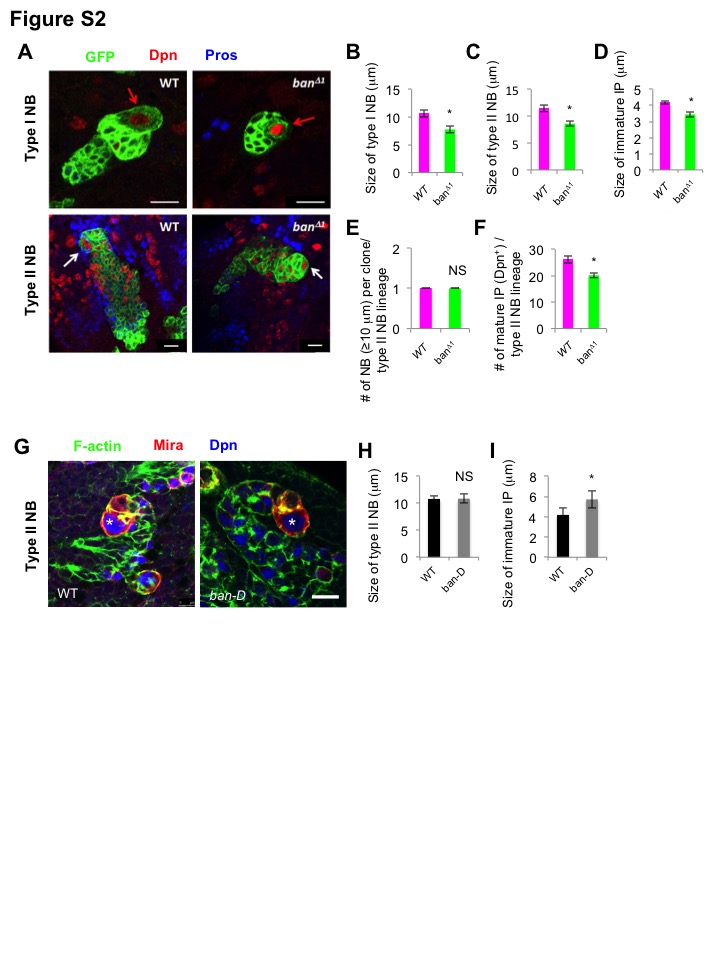

Supplement: S2 Fig — (A) MARCM analysis of type I and type II NBs in WT or banΔ1 mutant clones at 120 h ALH. Clones are labeled with GFP in green; type I and II NBs are marked with red and white arrows, respectively. (B, C) Quantification of cell sizes of type I (B) or type II (C) NBs in WT and banΔ1 mutant clones from A. *, p<0.005; n = 5–8 clones. (D) Quantification of cell size of immature IPs WT or banΔ1 mutant type II NB clones from A. *, p<0.005; n = 6 clones. (E) Quantification of NB number in WT or banΔ1 mutant type II NB clones. (NS) Not significant; n = 10 clones. (F) Quantification of the number of mature IPs in WT or banΔ1 mutant type II NB clones from A. *, p<0.005; n = 6 clones. (G) Effects of ban GOF (ban-D OE) on cell size in type II NBs or immature IPs. The type II NB lineages in late third instar larval brains of WT or ban-D OE animals driven by 1407-GAL4 are shown. Green: F-actin; Red: Miranda; Blue: Dpn; Asterisks: type II NBs. (H) Quantification of cell sizes of type II NBs in WT and ban-D OE brains from G. (NS) Not significant; n = 11 brains. (I) Quantification of cell sizes of immature IPs in WT and ban-D brains from G. *, p<0.001; n = 6 brains. Scale bars: A, G, 10 μm. (JPG) [file pgen.1006785.s003.jpg]

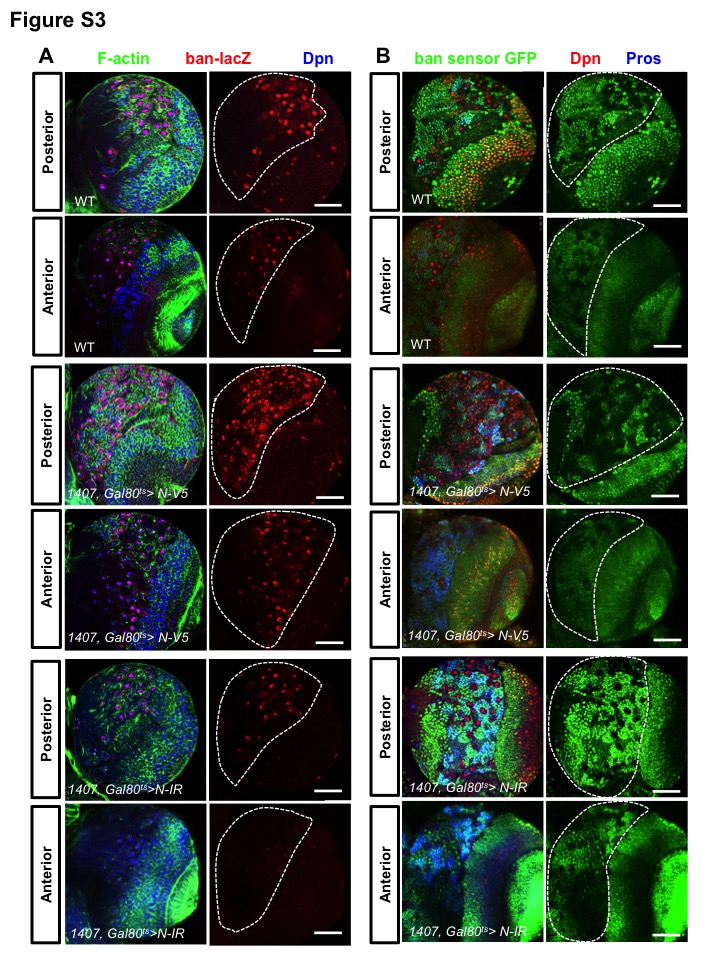

Supplement: S3 Fig — (A) Posterior and anterior views of ban-lacZ transcriptional reporter expression in immunostained WT, N-OE, or N RNAi (N-IR) brains. The 1407-GAL4, tub-GAL80ts system was used to induce N-V5 or N-IR transgene expression in both type I and type II NBs. White dashed lines outline the central brains. Zoomed in images and data quantification of ban GFP sensor expression in NBs are shown in Fig 3A and 3C. (B) Posterior and anterior views of ban GFP sensor expression in WT, N-OE, or N-IR brains. Zoomed in images and data quantification for LacZ expression in NBs is shown in Fig 3B and 3D. Scale bar in A, B: 50 μm. (JPG) [file pgen.1006785.s004.jpg]

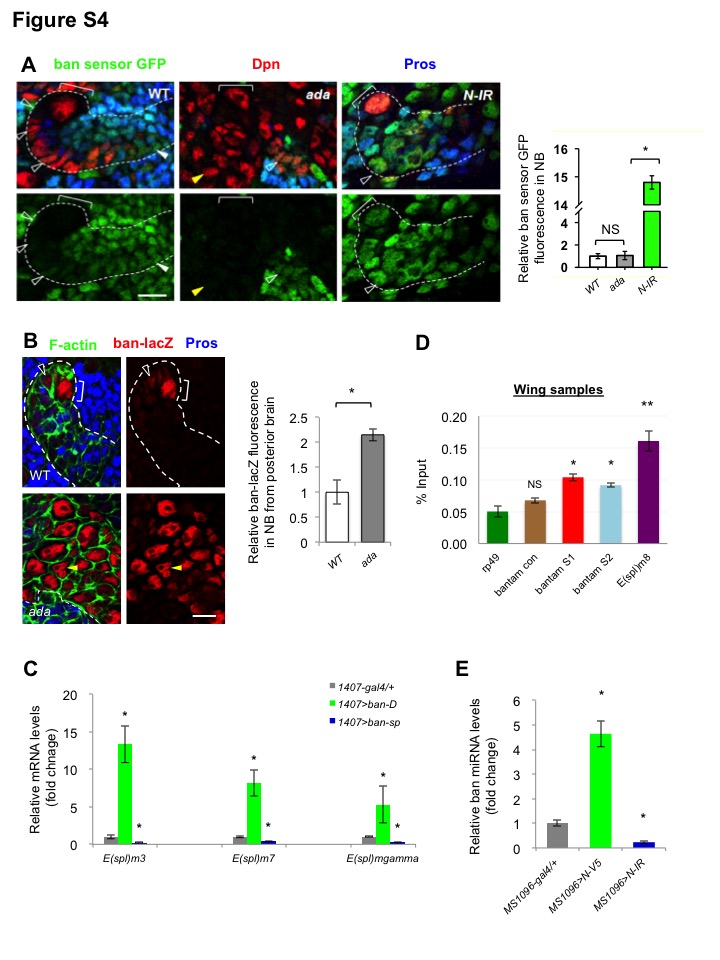

Supplement: S4 Fig — (A) Effects of α-ada LOF on ban activity as monitored with the GFP sensor. GFP sensor of ban was undetectable in ectopic type II NBs of α-ada homozygous mutants, whereas NBs with N RNAi (N-IR) driven by 1407-GAL4 showed low level ban GFP sensor expression. Green: GFP; Red: Dpn; Blue: Pros; Brackets: NBs. Bar graph shows quantification of ban sensor GFP fluorescence intensity in NB. *, p< 0.001, n = 12 brains. (B) Effects of α-ada LOF on ban-lacZ transcriptional reporter expression. Green: F-actin; Red: LacZ, Blue: Pros; Brackets: NBs. IPs in WT or α-ada mutant type II NB lineages are indicated with open or closed arrowheads, respectively. Bar graph shows quantification of LacZ immunofluorescence in type II NBs. *, P< 0.001, n = 12 brains. (C) ChIP analysis testing Su(H) binding to ban genomic DNA in wing discs. Quantitative PCR analysis revealed enrichment of ban sequences surrounding two putative Su(H)-binding sites (S1 and S2), but not a control ban sequence that does not contain a predicted Su(H)-binding site (ban con). See Fig 2F for positions of ban S1, S2, and con in the ban locus. E(spl)m8 and rp49 are positive and negative controls, respectively. **, p< 0.001, *, p< 0.05, n = 3 brains. (D) Quantitative RT-PCR analysis of mRNA levels of Notch target genes E(spl)m3, E(spl)m7 and E(spl)mγ in larval brains overexpressing ban (1407>ban-D) or ban-sp (1407>ban-sp). Data show the mean of 3 independent experiments after normalization with rp49. Error bars indicate s.e.m. *, p< 0.05. (E) Quantitative RT-PCR analysis of ban levels in wing discs. ban levels were normalized to 2S rRNA. *, p<0.01, n = 3 repeats. Scale bar: A, B, 10 μm. (JPG) [file pgen.1006785.s005.jpg]

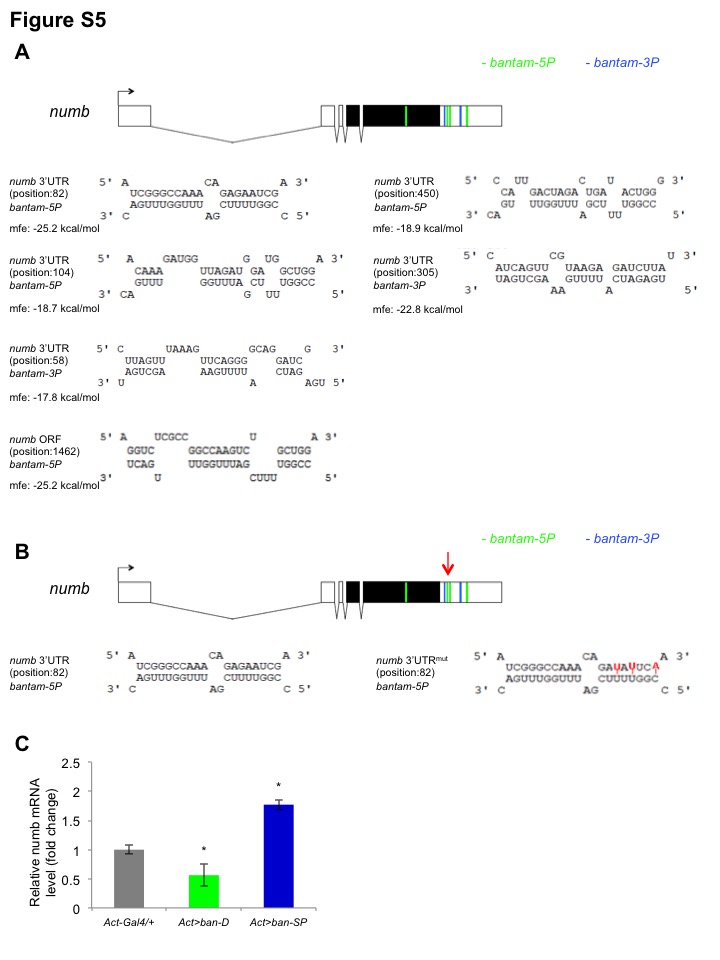

Supplement: S5 Fig — (A) Schematic of numb locus showing genomic organization and predicted target sites for ban-5p and ban-3p. ORF is indicated in black and UTR sequences in white. Sequences of predicted base-pairings between ban-5p or ban-3p and numb mRNA were identified in ORF and 3'UTR using the RNAHybrid program available at [http://bibiserv.techfak.uni-bielefeld.de/rnahybrid/submission.html]. Numbering is relative to first nucleotide of numb 3’UTR or ORF. Free energies for binding between ban miRNA and each target sites are listed. (B) Mutagenesis of numb ‘UTR for luciferase reporter assay. Left: The red arrow indicates the location of one predicted ban-5P target site in numb 3'UTR (position at 82) used for mutagenesis to generate RL-numb 3'UTRmut construct in Fig 4A. Right: Red labeled nucleotides indicate mutations introduced in the RL-numb 3'UTRmut construct. (C) Quantitative RT-PCR analysis of numb mRNA levels in ban (Act>ban-D) or ban-sp (Act>ban-sp) overexpressing larvae. The qRT-PCR analysis is correlated with the data shown in Fig 4B. Data show the mean of 3 independent experiments after normalization with rp49. Error bars indicate s.e.m. *, p< 0.05. (JPG) [file pgen.1006785.s006.jpg]

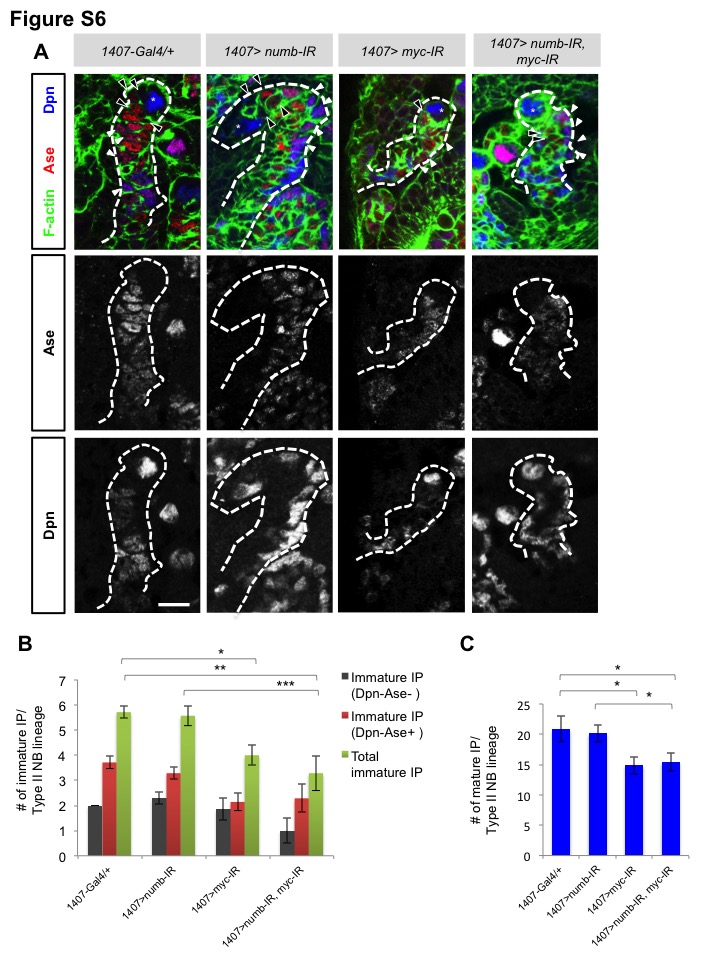

Supplement: S6 Fig — (A) The reduction of IP number and type II NB lineage size from myc RNAi, but no loss or cell fate transformation from numb RNAi driven by 1407-Gal4. At 120 h ALH, the type II NB lineages in larval central brains were analyzed. numb and myc double RNAi behaves similar phenotypes as myc RNAi. Type II NB lineages are marked with white dashed lines. Asterisks: type II NBs; open arrowheads: immature IPs; white arrowheads: mature IPs. Green: F-actin; Red: Ase; Blue: Dpn. (B) Quantification of number of immature IP from A. *, p<0.05; **, p<0.01; ***, p<0.001. n = 7 brains. (C) Quantification of number of mature IP from A. *, p<0.05, n = 7 brains. Scale bars: A, 10 μm. (JPG) [file pgen.1006785.s007.jpg]

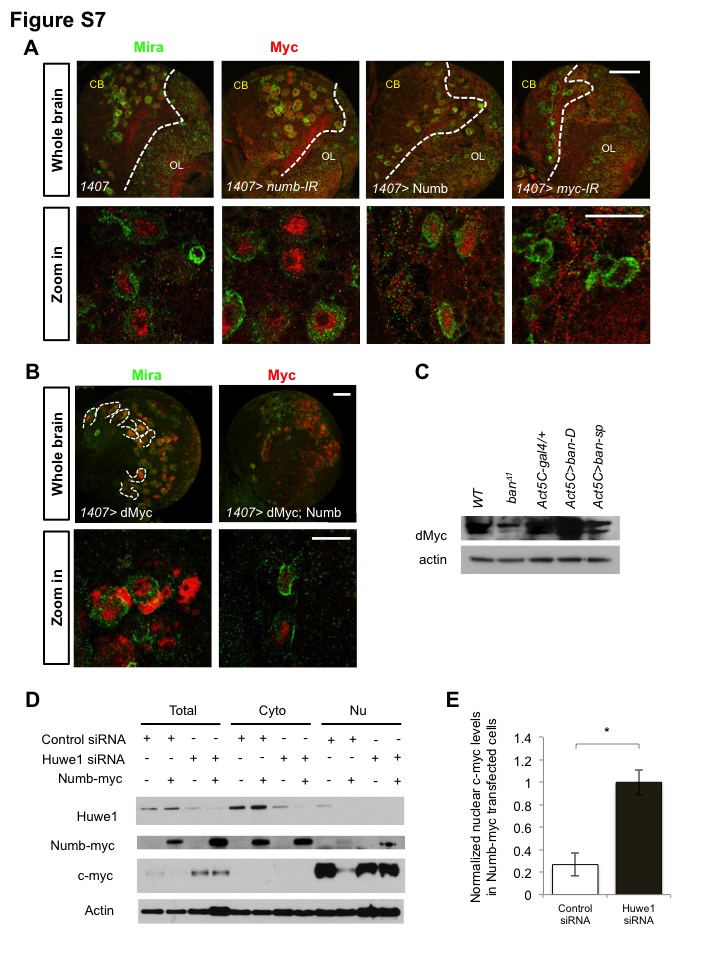

Supplement: S7 Fig — (A) Effects of Numb RNAi and Numb OE on Myc protein expression. Top panels: Posterior surface views of whole brains are shown. 1407/+, 1407>numb-IR, 1407>Numb, and 1407>myc-IR were immunostained at 120 h ALH for dMyc. White dotted line indicates the boundary between optical lobe (OL) and central brain (CB) regions. Bottom panels: zoomed in images of dMyc staining in type I NBs in top panels. Note that dMyc expression in the nucleus is abolished in 1407>myc-IR brain. Green: Miranda; Red: dMyc. (B) Supporting evidence that Numb OE attenuates Myc protein expression. Top panels: Whole brains of 1407> dMyc and 1407> dMyc; Numb immunostained at 120 h ALH for dMyc. Type II NB lineages are outlined. Bottom panels: zoom in images of Myc staining in NBs. Note that UAS-Numb transgene expression driven by 1407-GAL4 resulted in loss of type II NB lineages. Green: Miranda; Red: dMyc. (C) Western blot analysis of larval brain extracts showing effects of ban LOF or GOF on Numb protein levels. Actin serves as loading control. (D) Western blot analysis assessing the effect of Huwe1 RNAi on c-Myc protein level reduction caused by Numb overexpression in HEK293T cells. Cells with or without myc-tagged Numb (Numb-myc) expression and co-transfected with Huwe1 siRNA or control siRNA were fractionated and subjected to western blot analysis with the indicated antibodies. Efficient knockdown of Huwe1 by siRNA was revealed by anti-Huwe1 western blot. Total: total lysate; Cyto: cytosol fraction; Nu: nuclear fraction. (E) Quantification of normalized nuclear c-Myc levels in cells expressing Numb-myc vs. cells not expressing Numb-myc from E by comparing c-Myc levels after normalization with Actin. *, p<0.01. n = 3 independent experiments. Scale bars: A, C, 50 μm (top panels) and 20 μm (bottom panels). (JPG) [file pgen.1006785.s008.jpg]

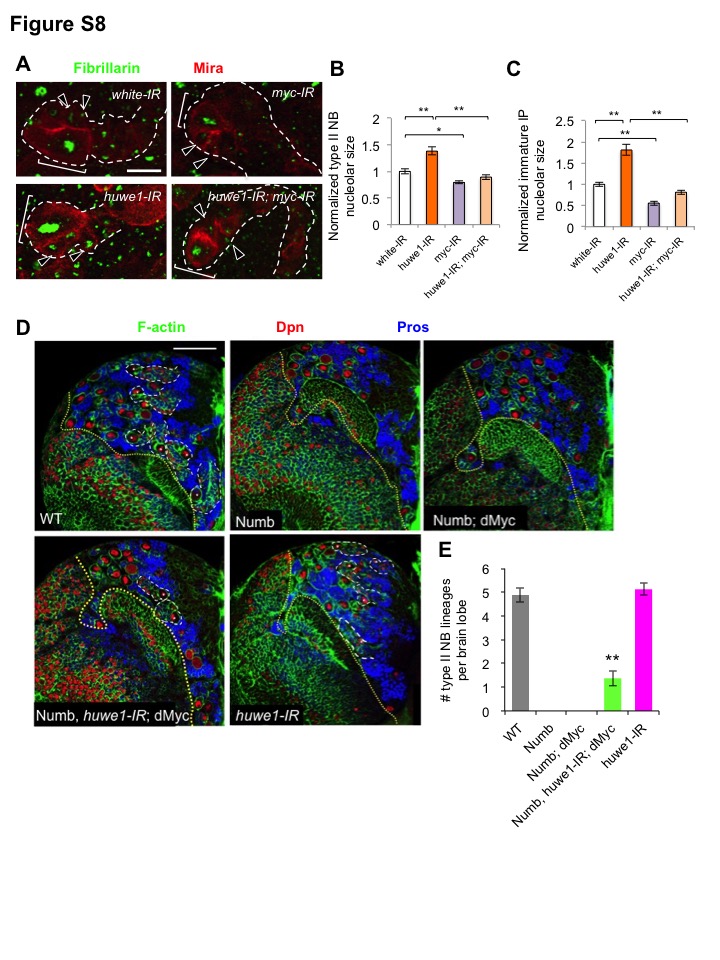

Supplement: S8 Fig — (A-C) Genetic interaction between huwe1 and myc in nucleolar size regulation. Larval brain expression of transgenes was driven by 1407-Gal4. Green, Fibrillarin; Red, Miranda. Brackets: type II NBs. Nucleoli of immature IPs are indicated by arrowheads. (B) Quantification of nucleolar size in type II NBs from A. **, p< 0.0001; n = 7 brains. (C) Quantification of nucleolar size in immature IP of type II NB lineages from A. *, p< 0.01; **, p< 0.0001; n = 7 brains. (D) Huwe1 RNAi promotes the ability of Myc OE to rescue the type II NB loss caused by Numb OE. Single optical section of type II NB lineages in WT, 1407>Numb, 1407>Numb; dMyc, 1407>Numb, huwe1 RNAi; dMyc, and 1407> huwe1 RNAi larval brains marked with white dashed lines were immunostained for NBs (Dpn), differentiated cells (Pros), and cell cortex (F-actin). Each type II NB expressing Dpn+ is indicated by a star. Yellow dotted line indicates the boundary between the optical lobe (left) and the central brain (right) region. Co-expression of Dicer2 was applied in all genotypes to enhance RNAi effect. (E) Quantification of number of type II NBs from D. **, p< 0.01 (vs Numb; dMyc); n = 8 brains. Scale bars: A, 10 μm; D, 50 μm. (JPG) [file pgen.1006785.s009.jpg]

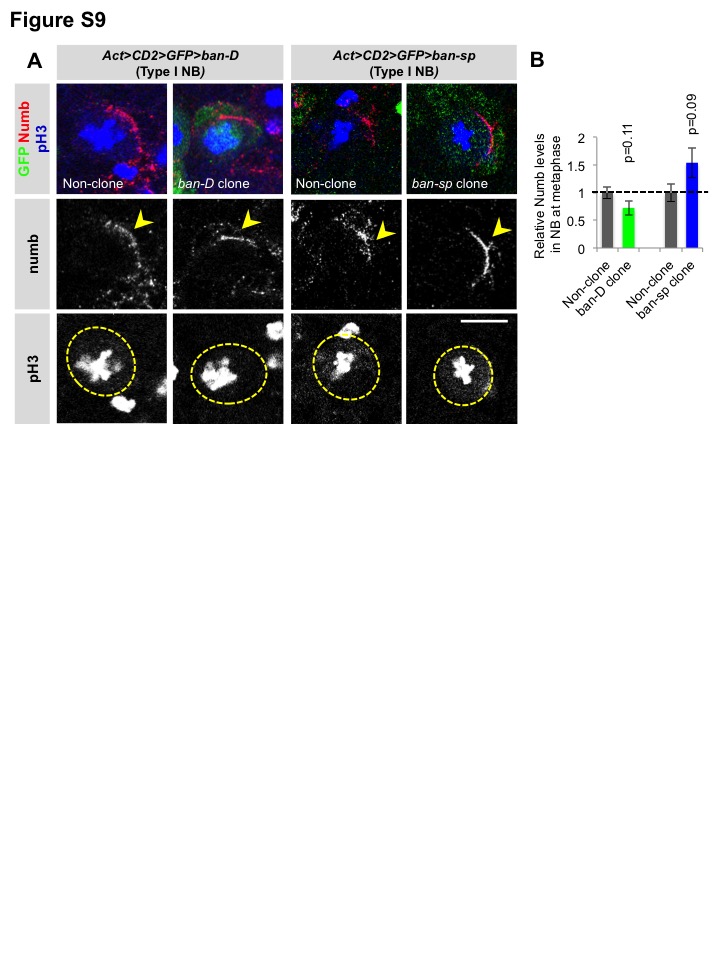

Supplement: S9 Fig — (A) Immunostaining of Numb expression in flip-out ban GOF (ban-D) and ban LOF (ban-sp) clones in larval brains. Clones and non-clones in the same brains were distinguished by the expression of the GFP marker and the type I NB lineages are outlined with yellow dashed line. Green: GFP; Red: Numb; Blue: pH3; Yellow arrowheads: Numb expression located at the basal side of NB cortex. (B) Quantification of Numb expression of type I NBs from A. n = 5–8 brains. Scale bars, 10 μm. (JPG) [file pgen.1006785.s010.jpg]

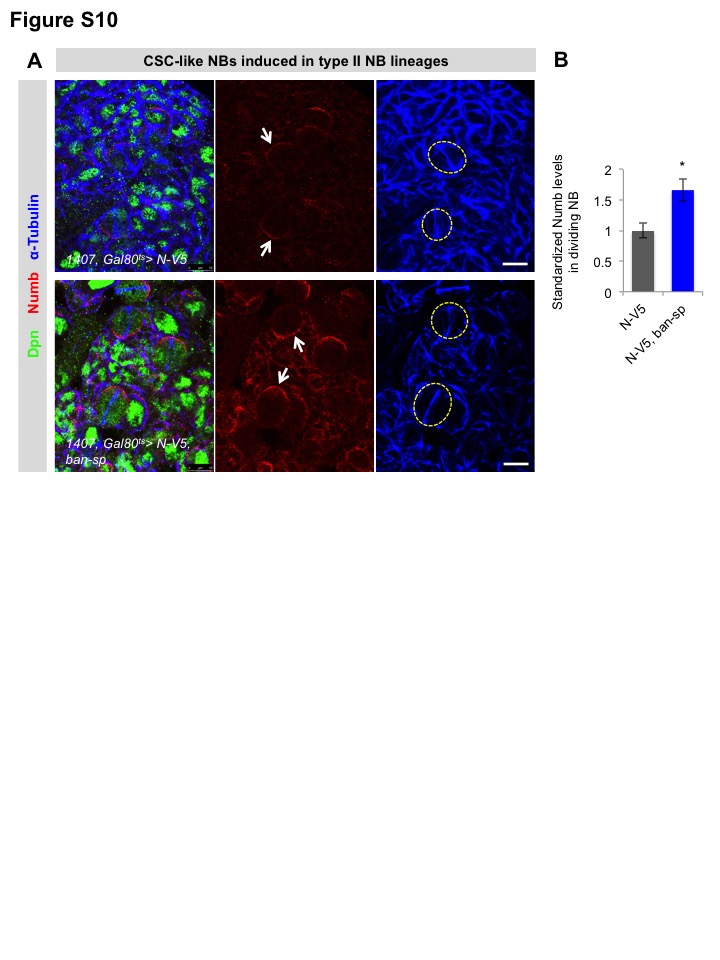

Supplement: S10 Fig — (A) Immunostaining of Numb protein in N-V5 (1407-Gal4:Gal80ts>N-V5) larval brains with or without ban-sp co-expression. Arrows indicate dividing NBs stained for α-Tubulin in blue and asymmetrically localized crescent-shaped Numb protein in red. (B) Quantification of the fluorescence intensity of Numb protein in dividing NBs from A. The analysis was done by measuring Numb protein immunofluorescence in the area with asymmetrically localized Numb protein crescent and normalize that with α-Tubulin levels. *, p<0.01, n = 5 brains. Scale bar: A, 10 μm. (JPG) [file pgen.1006785.s011.jpg]

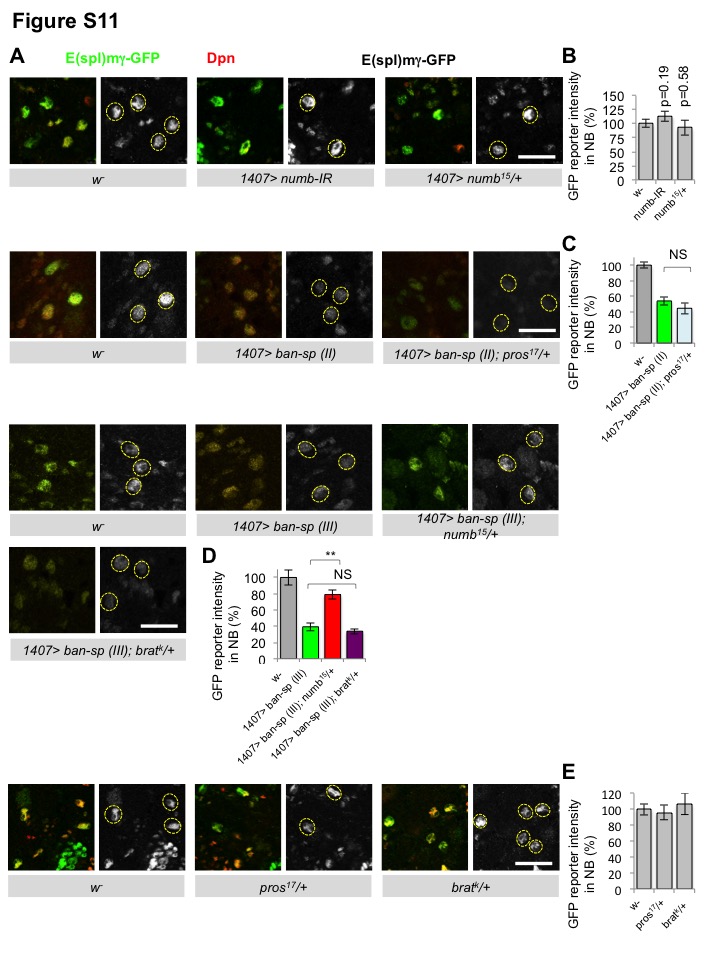

Supplement: S11 Fig — (A) The effects of pros, numb, or brat gene dosage on ban-sp induced reduction of E(spl)mγ-GFP reporter expression. Yellow dashed circle marks type I NBs located in the posterior brain with E(spl)mγ-GFP reporter expression. (B-E) Quantification of the fluorescence intensity of E(spl)mγ-GFP reporter in type I NBs from A. Expression of the reporter is not altered by numb RNAi or loss of one copy of numb as in numb15/+ condition in an otherwise wild type background. (B) In ban-sp overexpressing larval brain, E(spl)mγ-GFP reporter expression is sensitive to the loss of one copy of numb, but not brat (D) or pros (C). ban-sp (II) and ban-sp (III) in A indicate ban-sp transgenes located on the II or III chromosomes. **, p<0.0001 comparing 1407>ban-sp (III) with or without loss of one copy of numb. n = 6–12 brains in B-D. (E) Loss of one copy of pros or brat has no effect on E(spl)mγ-GFP reporter expression in type I NBs. n = 6 brains. Scale bars: A, 20 μm. (JPG) [file pgen.1006785.s012.jpg]

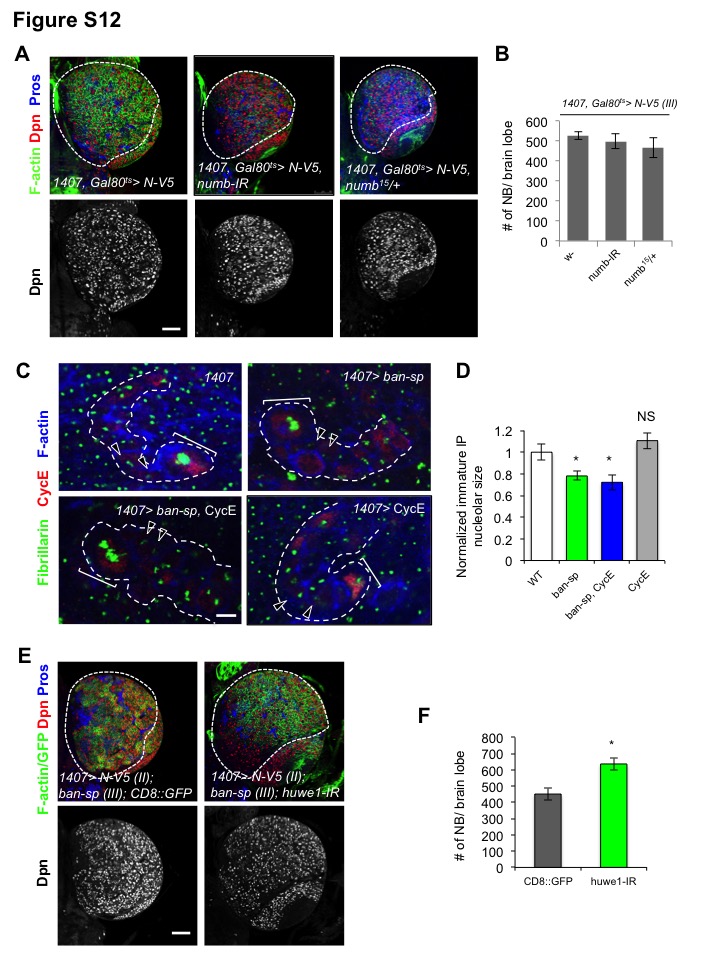

Supplement: S12 Fig — (A) numb RNAi or heterozygosity alone has no effect on N-induced brain tumor growth. Larval brains at 120h AHL were stained for Dpn (NBs), Pros (differentiated cells), and F-actin (cell cortex). (B) Quantification of total number of NBs is shown in A. (C) Overexpression of the cell cycle regulator CycE did not alter the effect of ban-sp overexpression in reducing nucleolar size of IPs in type II NBs. Green: Fibrillarin; Red: CycE; Blue: F-actin; Brackets: NBs. Arrowheads: nucleoli of immature IPs. (D) Quantification of nucleolar size of immature IPs in type II NB lineages from C. Transgenes were driven by the NB-specific 1407-Gal4. *, p< 0.005. n = 8 brains. (E) Effect of Huwe1 inhibition by RNAi on the phenotypes caused by ban-sp overexpression in N-induced brain tumor growth. Green: F-actin or GFP; Red: Dpn, Blue: Pros. (F) Quantification of the number of NBs shown in E. N-V5 (II) and ban-sp (III) in E indicate transgenes located on the II or III chromosomes. *, p< 0.005. n = 8 brains. Scale bars: A, E, 50 μm; C, 5 μm. (JPG) [file pgen.1006785.s013.jpg]
