# Supplementary material for: The bantam microRNA acts through Numb to exert cell growth control and feedback regulation of Notch in tumor-forming stem cells in the Drosophila brain
Source: PLoS Genet. 2017 May 17;13(5):e1006785. doi: 10.1371/journal.pgen.1006785 (PMC5453605; doi:10.1371/journal.pgen.1006785)
Supplement: S1 Table — (DOCX) [file pgen.1006785.s014.docx]

**Table S1**

| ***Gene*** | **Primer labeling in** **Fig 2F** & **Fig S3C** | **Sequence (5’ to 3’)** | **Putative Su(H) binding sites covered** |
| --- | --- | --- | --- |
| *E(spl)m8*-*HLH* | *E(spl)m8* | F: GGAGCGGAGCGAAGCATGTG  R: GCGTGGGAACCGAGCTGAAA | GTGAGAA |
| *bantam* | *bantam S1* | F: GTTCTTCGCTTCTCTGTGGTCT  R: ACCGGTTTCGTAGTCAAATCAT | ATGTGAG |
| *bantam* | *bantam S2* | F: ATTGGAGAAATACGGGCGC  R: TATTTTCACACGCGCCGC | GTGTGAA |
| *bantam* | *bantam con* | F: CCAATGCAATTGGAGAAATACGG  R: ACACAATTAGTGGCGCTGAG | - |
| *RP49 (RpL32)* | *rp49* | F: CCCTGATAAGGATTGATGCTG  R: CAGACCATTGCGGTCTTTCT | - |
