# Supplementary material for: The bantam microRNA acts through Numb to exert cell growth control and feedback regulation of Notch in tumor-forming stem cells in the Drosophila brain
Source: PLoS Genet. 2017 May 17;13(5):e1006785. doi: 10.1371/journal.pgen.1006785 (PMC5453605; doi:10.1371/journal.pgen.1006785)
Supplement: S2 Table — (DOCX) [file pgen.1006785.s015.docx]

**Table S2**

| **Gene & Primer labeling** | **Primer sequence (5’ to 3’)** | **Product size** |
| --- | --- | --- |
| *RP49 (RpL32)* | F: AGGGTATCGACAACAGAGTG  R: CACCAGGAACTTCTTGAATC | 122 bp |
| *E(spl)m3* | F: CGAGCCAGGATCAACAAGTG  R: CGGTCAACTCCAGGATATCC | 115 bp |
| *E(spl)m7* | F: GTTTCCGTGCTGGATACATC  R: CTGCTCCAGTTGGTTGAGAC | 131 bp |
| *E(spl)mgamma* | F: ATTTCGCCAATCTCCAGCTA  R: GGAGTCGACTGGTGGAAATC | 239 bp |
| *Numb* | F: GCTCCTTTCGCGATTCCTTT  R: AAGACCTTGAGTGCCTCCTC | 187 bp |
